# Socioeconomic, Clinical, and Molecular Features of Breast Cancer Influence Overall Survival of Latin American Women

**DOI:** 10.3389/fonc.2022.845527

**Published:** 2022-03-08

**Authors:** Liz Maria de Almeida, Sandra Cortés, Marta Vilensky, Olivia Valenzuela, Laura Cortes-Sanabria, Mirian de Souza, Rafael Alonso Barbeito, Eliana Abdelhay, Nora Artagaveytia, Adrian Daneri-Navarro, Andrea S. Llera, Bettina Müller, Osvaldo L. Podhajcer, Carlos Velazquez, Elsa Alcoba, Isabel Alonso, Alicia I. Bravo, Natalia Camejo, Dirce Maria Carraro, Mónica Castro, Sandra Cataldi, Alfonso Cayota, Mauricio Cerda, Alicia Colombo, Susanne Crocamo, Alicia Del Toro-Arreola, Raul Delgadillo-Cristerna, Lucia Delgado, Marisa Dreyer Breitenbach, Elmer Fernández, Jorge Fernández, Wanda Fernández, Ramon A. Franco-Topete, Fancy Gaete, Jorge Gómez, Leivy P. Gonzalez-Ramirez, Marisol Guerrero, Susan A. Gutierrez-Rubio, Beatriz Jalfin, Alejandra Lopez-Vazquez, Dora Loria, Silvia Míguez, Andres de J. Moran-Mendoza, Gilberto Morgan-Villela, Carina Mussetti, Maria Aparecida Nagai, Antonio Oceguera-Villanueva, Rui M. Reis, Javier Retamales, Robinson Rodriguez, Cristina Rosales, Efrain Salas-Gonzalez, Laura Segovia, Juan M. Sendoya, Aida A. Silva-Garcia, Stella Viña, Livia Zagame, Beth Jones, Moysés Szklo, United States-Latin American Cancer Research Network (US-LACRN), Juan Abarca

**Affiliations:** ^1^ Instituto Nacional de Câncer, Rio de Janeiro, Brazil; ^2^ Pontificia Universidad Católica de Chile, Santiago, Chile; ^3^ Instituto de Oncología Angel Roffo, Buenos Aires, Argentina; ^4^ Universidad de Sonora, Hermosillo, Mexico; ^5^ Hospital de Especialidades, CMNO-IMSS, Guadalajara, Mexico; ^6^ Facultad de Medicina, Montevideo, Uruguay; ^7^ Hospital de Clínicas Manuel Quintela, Universidad de la República, Montevideo, Uruguay; ^8^ Universidad de Guadalajara, Guadalajara, Mexico; ^9^ Fundación Instituto Leloir, CONICET, Buenos Aires, Argentina; ^10^ Instituto Nacional del Cáncer, Santiago, Chile; ^11^ Hospital Municipal de Oncología María Curie, Buenos Aires, Argentina; ^12^ Centro Hospitalario Pereira Rossell, Montevideo, Uruguay; ^13^ Hospital Regional de Agudos Eva Perón, Buenos Aires, Argentina; ^14^ AC Camargo Cancer Center, São Paulo, Brazil; ^15^ Instituto Nacional de Cáncer, Montevideo, Uruguay; ^16^ Institut Pasteur de Montevideo, Montevideo, Uruguay; ^17^ Universidad de Chile, Santiago, Chile; ^18^ Universidade do Estado do Rio de Janeiro, Rio de Janeiro, Brazil; ^19^ Universidad Católica de Córdoba, Centro de Investigaciones en Bioquímica Clínica e Inmunologia-CONICET, Córdoba, Argentina; ^20^ Instituto de Salud Pública, Santiago, Chile; ^21^ Hospital San Borja Arriarán, Santiago, Chile; ^22^ OPD Hospital Civil de Guadalajara, Universidad de Guadalajara, Guadalajara, Mexico; ^23^ Hospital Luis Tisne, Santiago, Chile; ^24^ Texas A&M University, Houston, TX, United States; ^25^ Hospital San José, Santiago, Chile; ^26^ Hospital de Gineco-Obstetricia CMNO-IMSS, Guadalajara, Mexico; ^27^ Registro Nacional de Cancer, Montevideo, Uruguay; ^28^ Instituto de Câncer de São Paulo, São Paulo, Brazil; ^29^ Instituto Jalisciense de Cancerologia, Guadalajara, Mexico; ^30^ Hospital de Câncer de Barretos, Barretos, Brazil; ^31^ Grupo Oncológico Cooperativo Chileno de Investigación, Santiago, Chile; ^32^ Hospital Central de las Fuerzas Armadas, Montevideo, Uruguay; ^33^ Hospital Barros Luco Trudeau, Santiago, Chile; ^34^ Yale School of Public Health, Yale University, New Heaven, CT, United States; ^35^ Johns Hopkins Bloomberg School of Public Health, Johns Hopkins University, Baltimore, MD, United States

**Keywords:** global excellence, oncology, Latin America, breast cancer, molecular subtypes, risk factors, prognosis

## Abstract

Molecular profile of breast cancer in Latin-American women was studied in five countries: Argentina, Brazil, Chile, Mexico, and Uruguay. Data about socioeconomic characteristics, risk factors, prognostic factors, and molecular subtypes were described, and the 60-month overall cumulative survival probabilities (OS) were estimated. From 2011 to 2013, 1,300 eligible Latin-American women 18 years or older, with a diagnosis of breast cancer in clinical stage II or III, and performance status ≦̸1 were invited to participate in a prospective cohort study. Face-to-face interviews were conducted, and clinical and outcome data, including death, were extracted from medical records. Unadjusted associations were evaluated by Chi-squared and Fisher’s exact tests and the OS by Kaplan–Meier method. Log-rank test was used to determine differences between cumulative probability curves. Multivariable adjustment was carried out by entering potential confounders in the Cox regression model. The OS at 60 months was 83.9%. Multivariable-adjusted death hazard differences were found for women living in Argentina (2.27), Chile (1.95), and Uruguay (2.42) compared with Mexican women, for older (≥60 years) (1.84) compared with younger (≤40 years) women, for basal-like subtype (5.8), luminal B (2.43), and HER2-enriched (2.52) compared with luminal A subtype, and for tumor clinical stages IIB (1.91), IIIA (3.54), and IIIB (3.94) compared with stage IIA women. OS was associated with country of residence, PAM50 intrinsic subtype, age, and tumor stage at diagnosis. While the latter is known to be influenced by access to care, including cancer screening, timely diagnosis and treatment, including access to more effective treatment protocols, it may also influence epigenetic changes that, potentially, impact molecular subtypes. Data derived from heretofore understudied populations with unique geographic ancestry and sociocultural experiences are critical to furthering our understanding of this complexity.

## Introduction

Breast cancer is the leading cause of death from cancer in women worldwide and in Latin America (1). Breast tumors can be stratified by histological and immunopathological as well as by molecular subtypes (2). Socioeconomic and cultural-mediated factors contribute to breast cancer heterogeneity survival (3–6). Advanced stage at diagnosis, high grade, negative hormone receptors, and HER-2 amplification have been found to be associated with higher breast cancer mortality (7).

Studies of Latin American women have shown differences in the distribution of molecular subtypes of breast cancer by geographic regions (8) which reflect differences in genetic background of the population (9). Data from heretofore understudied populations with unique geographic ancestry and sociocultural experiences are critical to furthering our understanding of this population heterogeneity (10).

Latin American women have a lower incidence but higher mortality from breast cancer than those observed in women from developed countries, except in Argentina and Uruguay, where the incidences are higher than in Latin America countries (**Supplementary Figure S1**) (11, 12). Estimates of breast cancer survival in 2010–2014 were 84.4%, 75.5%, and 75.2% for Argentina, Chile, and Brazil (13).

To better understand the characteristics and survival of Latin American women with breast cancer, epidemiologists from Argentina, Brazil, Chile, Mexico, Uruguay, and the USA conducted the Molecular Profiling of Stage II and III Breast Cancer in Latin American Women Receiving Standard-of-Care Treatment (MPBC) study, proposed by the US-Latin America Cancer Research Network (US-LACRN) (14). The goal of this study was to describe the characteristics of the Latin American women with breast cancer by country and to identify predictors for 60-month cumulative probability of overall survival. To our knowledge, this is the first comprehensive, multisite study focusing on Latin American breast cancer patients including molecular, clinical, and epidemiological profiles to overall (all-cause) survival.

## Material and Methods

### Study Design

Between 2011 and 2013, a prospective cohort study was conducted in Argentina, Brazil, Chile, Mexico, and Uruguay, where eligible women with recently diagnosed breast cancer were recruited from reference cancer treatment centers. Using standardized questionnaire, trained interviewers collected epidemiological data. In addition to molecular profiling of tumor tissue described in an earlier publication (14), clinical data were collected from medical records. Women were followed up through death from any cause for 5 years (**Supplementary Figure S2**).

### Settings

Three hospitals in Argentina (two in the Autonomous City of Buenos Aires and one in Buenos Aires Province), four in Brazilian cities (one in Rio de Janeiro, two in São Paulo, and one in Barretos), eleven in Mexico cities (six in Hermosillo, one in Obregón, and four in Guadalajara), four in Chile in the Metropolitan Area of Santiago, and four in Montevideo (Uruguay) were included.

### Inclusion and Exclusion Criteria

Eligible Latin American women who were ≥18 years old, reside in the recruiting locations, looking for treatment in the selected hospitals, with a new confirmed histopathological diagnosis of breast cancer, clinical stage II or III, and performance status (PS) ≦̸1were invited to participate in the study and, after signing the consent form, were included. Exclusion criteria were prior history of nonbreast malignancy in the previous 5 years, *in situ*, bilateral invasive or inflammatory breast cancer, and clinical or radiological evidence of distant metastases. Pregnant or lactating women were also ineligible.

### Data Collection

The recruitment period in the selected hospitals began in November 2011 and finished in December 2013, including 1,300 women. After signing the consent form, the women were interviewed by nurses in private settings, using an instrument that included questions of socioeconomic and demographic characteristics and lifestyle factors (age, education, alcohol and tobacco use, physical activity, and Body Mass Index), as well as access to healthcare, ancestry, familial cancer, hormonal and reproductive history, medical history, anthropometric data, alcohol intake, tobacco use, and physical activity (**Supplementary Material 3**). The questionnaire and its instruction manual were translated to English, Spanish, and Portuguese. All versions were cross-compared. Data were stored in Open Clinica^®^ (openclinica.com) and were managed by WESTAT under NCI contract. Clinical-pathological and treatment information registered in CRFs included age, histological type, histological grade, clinical stage, lymph node status, tumor size at diagnosis, estrogen receptor (ER), progesterone receptor (PgR), human epidermal growth factor 2 (HER2) and Ki67 expression, neoadjuvant therapy, and adjuvant therapy. Researchers trained by oncologists abstracted clinical and outcome data, including information on vital status and date of death, during the follow-up. Data managers in each site followed the standardized protocol reviewing and coding free text and notes before data entry and conducting quality checks following data entry. After clinical evaluation, patients were treated according to standard of care of each institution in each country.

### Biological Tissue Collection

All sample collection procedures were carried out according to the LACRN consensus standard operating procedures for frozen and formalin-fixed, paraffin-embedded (FFPE) specimens based on the TCGA best practice recommendations (https://brd.nci.nih.gov/brd/sop-compendium/show/701). Tissue samples were obtained from core needle biopsies or from surgical resections.

### PAM50 Subtype Assignation

PAM50 subtype assignation was performed with gene expression data obtained from two-color Agilent 4x44k v2 microarrays (15). A training set of balanced ER^−^ and ER^+^ cases sampled from all countries was used for centering the gene expression data of the whole cohort, and the PAM50 algorithm was run as described elsewhere (https://genome.unc.edu/pubsup/breastGEO/PAM50.zip). Each site performed gene expression analysis independently with their own equipment, after a thorough harmonization of all the molecular aspects related to sample obtention, storage, RNA extraction, and microarray analysis.

### Data Analysis

From the 1,300 patients of the cohort, 1,140 answered the epidemiological questionnaire. From these, 177 patients were excluded from the analysis due to lack of gene expression and PAM50 data, and 64, due to lack of clinical staging. The analytic sample of this epidemiological study consisted of 863 breast cancer patients.

The sociodemographic variables were described as follows: (a) Age: less than 40 years, 40–49, 50–59, and 60 years and over; (b) education: 0–8 completed years, 9–24, and unknown; and (c) Body Mass Index: <25.0, 0–29.99 kg/m^2^, ≥30.0 kg/m^2^, and unknown. Lifestyle variables: alcohol use: none, any, and unknown; tobacco use: never smoke, smoker, ex-smoker, and unknown; and physical activity: no, yes, and unknown. Reproductive, hormonal, and breast cancer history variables: age range at menarche: ≤12 years and ≥13 years and over; use of oral contraceptives: ever use vs. never use; at least one full-term pregnancy, breastfeeding, hormone replacement therapy, self-reported previous benign breast diseases, and first-degree family history of breast cancer: yes vs. no; menopause status: premenopausal vs. postmenopausal; and type of menopause: natural causes vs. surgery or other reasons.

Molecular and clinical characteristics of the tumor were classified according to the PAM50 in: luminal A, luminal B, HER2-enriched, basal-like, and normal-like subtypes. Gene expression distribution of PAM50 genes in all samples were previously tested by principal component analysis to confirm that there were no associations with potential sources of bias such as country, arm (neoadjuvant or adjuvant therapy), processing date, or sample quality. Clinical stage at diagnosis was stratified as IIA, IIB, IIIA, and IIIC.

Unadjusted associations between country of residence and selected characteristics were evaluated by Chi-squared and Fisher’s exact tests (*p* < 0.05). Overall 60-month cumulative survival probability (OS) was estimated by the Kaplan–Meier method. Follow-up started with the informed consent form signature. Women were followed up through death, loss to follow-up, or end of the study. Cumulative probabilities based on less than 10 patients, e.g., HER2-enriched and basal-like subtypes in Uruguay were not examined. The log-rank test was used to determine the differences between the estimated cumulative probability curves. Variables with *p*-values <0.20 were entered in the multivariable-adjusted Cox proportional hazards model (adjusted by country of residence, age, education, PAM50 intrinsic subtype, and tumor clinical stage). The proportional-hazards assumption over time was evaluated by a test based on Schoenfeld residuals. Stata 15 was used to perform all analyses (16).

### Ethics Issues

Local ethics committee of the institutions of each country (18 centers) and the National Cancer Institute ethics committee approved this study (**Supplementary Material 4**). All participants who agreed to participate signed informed consent forms.

## Results

### General and Between-Country Differences in Breast Cancer Risk Factors

The majority (65%) of Latin American women were aged 50 years and over (median = 54 years old, 1st quartile = 45, and 3rd quartile = 62) and never used alcohol (63%) or tobacco (57%). Fifty-two percent were not physically active and 66% were overweight. Differences among the country’s distributions were statistically significant (*p*-value <0.05) for age group, education level, alcohol and tobacco use, physical activity but not for body mass index (**Table 1**). Uruguay’s women were older than those from other countries. Chile’s women had a higher proportion of completed years of schooling compared with those from other countries. More than half of the women had ever used tobacco in Uruguay and Chile. In Uruguay and Brazil, women were found to drink alcohol beverages more often than those in other countries. Chile showed the highest proportion of smokers and physically inactive women (**Table 1**).

**Table 1:** Distribution of Latin American women with breast cancer by country of residence, according to sociodemographic and lifestyle factors.

Sociodemographic and lifestyle factors	Country of residence										Total		*p*-value
	Argentina		Brazil		Chile		Mexico		Uruguay				
	*n*	%	*n*	%	*n*	%	*n*	%	*n*	%	*n*	%	
Total	209	24.2	203	23.5	149	17.3	234	27.1	68	7.9	863	100.0	n.a.
Age group													
Less than 40 years	19	9.1	24	11.8	13	8.7	30	12.8	6	8.8	92	10.7	0.006
40 to 49 years	46	22.0	46	22.7	30	20.1	73	31.2	13	19.1	208	24.1	
50 to 59 years	71	34.0	76	37.4	48	32.2	73	31.2	15	22.1	283	32.8	
60 years and over	73	34.9	57	28.1	58	38.9	58	24.8	34	50.0	280	32.4	
Education													
0 to 8 years	105	50.2	114	56.2	54	36.2	122	52.1	41	60.3	436	50.5	0.003*
9 to 24 years	104	49.8	88	43.3	91	61.1	107	45.7	27	39.7	417	48.3	
Unknown	0	0.0	1	0.5	4	2.7	5	2.1	0	0.0	10	1.2	
Alcohol use													
None	126	60.3	98	48.3	99	66.4	189	80.8	30	44.1	542	62.8	<0.001*
Any	77	36.8	105	51.7	45	30.2	43	18.4	36	52.9	306	35.5	
Unknown	6	2.9	0	0.0	5	3.4	2	0.9	2	2.9	15	1.7	
Tobacco use													
Never smoke	117	56.0	126	62.1	61	40.9	162	69.2	29	42.6	495	57.4	<0.001*
Smoker	39	18.7	23	11.3	37	24.8	14	6.0	13	19.1	126	14.6	
Ex-smoker	50	23.9	54	26.6	50	33.6	57	24.4	24	35.3	235	27.2	
Unknown	3	1.4	0	0.0	1	0.7	1	0.4	2	2.9	7	0.8	
Physical activity													
No	86	41.1	101	49.8	85	57.0	96	41.0	32	47.1	400	46.3	0.014*
Yes	117	56.0	100	49.3	63	42.3	136	58.1	30	44.1	446	51.7	
Unknown	6	2.9	2	1.0	1	0.7	2	0.9	6	8.8	17	2.0	
Body Mass Index (BMI)													
<25.0 kg/m^2^	62	29.7	55	27.1	35	23.5	57	24.4	17	25.0	226	26.2	0.268*
25.0–29.99 kg/m^2^	71	34.0	72	35.5	57	38.3	88	37.6	15	22.1	303	35.1	
≥30.0 kg/m^2^	59	28.2	59	29.1	50	33.6	71	30.3	30	44.1	269	31.2	
Unknown	17	8.1	17	8.4	7	4.7	18	7.7	6	8.8	65	7.5	

^*^The p-value of the Chi-square test does not include the unknown category. n.a., Not applicable.

Among reproductive factors (**Table 2**), cross-country differences were statistically significant (*p* < 0.05) for oral contraceptive use, breastfeeding history, type of menopause, hormone replacement therapy (HRT), self-reported benign breast diseases, and first-degree breast cancer family history. Most women had menarche when older than 12 years and were postmenopausal when younger than 50 years, with 3/4 experiencing natural menopause. Oral contraceptives had been used by 60% of the women. At least 90.2% of the women had one or more full-term pregnancy and 80% had breastfed their children. The vast majority of women had not used hormone replacement therapy (86.7%). More than half reported benign breast diseases (57.9%) and only 7.3% had a first-degree family history of breast cancer.

**Table 2:** Distribution of Latin American women with breast cancer by country of residence, according to reproductive and hormonal characteristics, previous benign breast diseases, and first-degree family history of breast cancer.

Reproductive, hormonal, and breast cancer history	Country of residence										Total		*p*-value
	Argentina		Brazil		Chile		Mexico		Uruguay				
	*n*	%	n	%	*n*	%	*n*	%	*n*	%	*n*	%	
Age range at menarche													
≤12 years	93	44.5	93	45.8	67	45.0	96	41.0	39	57.4	388	45.0	0.161^*^
≥13 years and over	113	54.1	109	53.7	78	52.3	136	58.1	27	39.7	463	53.7	
Unknown	3	1.4	1	0.5	4	2.7	2	0.9	2	2.9	12	1.4	
Use of oral contraceptives													
Ever use	115	55.0	161	79.3	81	54.4	117	50.0	47	69.1	521	60.4	<0.001^*^
Never use	92	44.0	42	20.7	65	43.6	116	49.6	20	29.4	335	38.8	
Unknown	2	1.0	0	0.0	3	2.0	1	0.4	1	1.5	7	0.8	
At least 1 full-term pregnancy													
Yes	195	93.3	176	86.7	131	87.9	214	91.5	62	91.2	778	90.2	0.105^**^
No	10	4.8	25	12.3	13	8.7	19	8.1	5	7.4	72	8.3	
Unknown	4	1.9	2	1.0	5	3.4	1	0.4	1	1.5	13	1.5	
Breastfeeding													
Yes	172	82.3	153	75.4	123	82.6	182	77.8	60	88.2	690	80.0	0.006^**^
No^a^	29	13.9	48	23.6	21	14.1	50	21.4	5	7.4	153	17.7	
Unknown	8	3.8	2	1.0	5	3.4	2	0.9	3	4.4	20	2.3	
Menopause status													
Premenopausal	67	32.1	77	37.9	41	27.5	92	39.3	24	35.3	301	34.9	0.128^*^
Postmenopausal	140	67.0	124	61.1	106	71.1	140	59.8	40	58.8	550	63.7	
Unknown	2	1.0	2	1.0	2	1.3	2	0.9	4	5.9	12	1.4	
Age at menopause													
≤50 years	84	60.0	74	59.7	58	54.7	108	77.1	26	65.0	350	63.6	0.101^**^
≥51 years and over	35	25.0	26	21.0	9	8.5	27	19.3	10	25.0	107	19.5	
Unknown	21	15.0	24	19.4	39	36.8	5	3.6	4	10.0	93	16.9	
Type of menopause													
Natural causes	122	87.1	100	80.6	78	73.6	90	64.3	27	67.5	417	75.8	<0.001^*^
Surgery or other reasons	18	12.9	23	18.5	21	19.8	47	33.6	11	27.5	120	21.8	
Unknown	0	0.0	1	0.8	7	6.6	3	2.1	2	5.0	13	2.4	
Hormone replacement therapy													
Yes	12	5.7	37	18.2	29	19.5	22	9.4	4	5.9	104	12.1	
No	195	93.3	165	81.3	118	79.2	211	90.2	59	86.8	748	86.7	<0.001^**^
Unknown	2	1.0	1	0.5	2	1.3	1	0.4	5	7.4	11	1.3	
Self-reported previous benign breast diseases													
Yes	97	46.4	158	77.8	59	39.6	125	53.4	61	89.7	500	57.9	<0.001^**^
No	112	53.6	45	22.2	90	60.4	109	46.6	7	10.3	363	42.1	
First-degree family history of breast cancer													
Yes	18	8.6	19	9.4	2	1.3	17	7.3	7	10.3	63	7.3	<0.001^**^
No	191	91.4	184	90.6	147	98.7	217	92.7	61	89.7	800	92.7	

^*^The p-value of the Chi-square test does not include the category unknown.^**^The p-value of the Fisher’s test does not include the category unknown. ^a^Includes never pregnant women.

The most common tumors were luminal A (42.9%) and luminal B (21.4%) followed by basal-like (16.2%), HER2-enriched subtype (12.7%), and normal-like subtype (6.7%). Percentages of luminal A subtype varied from 36.8% in Mexico to 50.0% in Argentina and 50.2% in Uruguay. Uruguay also showed the highest proportion of luminal B (29.4%). HER2-enriched subtype was high in Mexico (17.1%) and low in Uruguay (8.8%). The highest variability between countries was found for basal-like breast cancer subtype: from 4.4% in Uruguay to 21.4% in Mexico. Fifty-eight breast tumors were classified as normal-like subtype according to PAM50 and the highest proportion was found in Argentina (8.6%). Seventy percent of Latin American women were diagnosed at stage II (38% at IIA, 32% at IIB) and 30% at stage III (20% at IIIA, 10% at IIIB). We observed some differences by country: 61% of Brazil’s and 84% of Uruguay’s women were diagnosed at stage II. Brazil had the highest percentage of women in stage IIIB: 21% (**Table 3**).

**Table 3:** Molecular characteristic of the tumor according to PAM50 intrinsic subtype and tumor clinical stage at diagnosis of Latin American women with breast cancer by country of residence.

Molecular and clinical characteristics of the tumor	Country of residence										Total		*p*-value
	Argentina		Brazil		Chile		Mexico		Uruguay				
	*n*	%	*n*	%	*n*	%	*n*	%	*n*	%	*n*	%	
PAM50 intrinsic subtype													
Luminal A	105	50.2	82	40.4	63	42.3	86	36.8	34	50.0	370	42.9	0.005
Luminal B	30	14.4	53	26.1	39	26.2	43	18.4	20	29.4	185	21.4	
HER2-enriched	24	11.5	24	11.8	16	10.7	40	17.1	6	8.8	110	12.7	
Basal-like	32	15.3	33	16.3	22	14.8	50	21.4	3	4.4	140	16.2	
Normal-like	18	8.6	11	5.4	9	6.0	15	6.4	5	7.4	58	6.7	
Clinical stage at diagnosis													
IIA	92	44.0	90	44.3	46	30.9	67	28.6	36	52.9	331	38.4	<0.001
IIB	60	28.7	35	17.2	58	38.9	100	42.7	21	30.9	274	31.7	
IIIA	43	20.6	35	17.2	35	23.5	55	23.5	6	8.8	174	20.2	
IIIB	14	6.7	43	21.2	10	6.7	12	5.1	5	7.4	84	9.7	

### Overall Cumulative Survival Probabilities for Latin American Women

The unadjusted OS at 60 months for all Latin American women was 83.9% (95% CI, 81.2–86.2). OS cumulative probabilities were similar among countries, ranging from 81% in Argentina to 88% in Mexico (**Table 4**).

**Table 4:** Overall cumulative survival probability at 60 months of Latin American women with breast cancer according to sociodemographic characteristics and lifestyle factors.

Sociodemographic and lifestyle factors	Cumulative survival probability at 60 months (PS60)		Log-rank *p*-value
	PS60	95% CI	
Global	83.9	(81.2–86.2)	n.a.
Country of residence			
Argentina	80.9	(74.4–85.9)	0.402
Brazil	84.0	(78.1–88.4)	
Chile	81.9	(74.7–87.2)	
Mexico	87.8	(82.6–91.5)	
Uruguay	82.9	(71.2–90.2)	
Age group			
Less than 40 years	83.7	(74.1–90.0)	0.799
40 to 49 years	85.2	(79.4–89.5)	
50 to 59 years	84.7	(79.8–88.5)	
60 years and over	82.2	(77.0–86.3)	
Education			
0 to 8 years	81.6	(77.5–85.0)	0.074
9 years or more	86.5	(82.7–89.5)	
Alcohol use			
None	85.4	(82.0–88.2)	0.248
Any	82.1	(77.1–86.1)	
Tobacco use			
Never smoke	85.2	(81.6–88.1)	0.372
Smoker	83.6	(75.5–89.2)	
Ex-smoker	80.9	(75.1–85.5)	
Physical activity			
No	83.8	(79.7–87.2)	0.864
Yes	84.1	(80.2–87.2)	
Body mass index			
<25.0 kg/m^2^	84.7	(79.1–89.0)	0.357
25.0–29.99 kg/m^2^	82.6	(77.7–86.5)	
≥30.0 kg/m^2^	86.8	(82.0–90.4)	

n.a., Not applicable.

Statistical differences (log-rank *p* < 0.05) were observed only for PAM-50 intrinsic subtypes and tumor stage (**Table 5**). For PAM50 intrinsic subtypes, the OS were estimated as 92% for luminal A, 80% for luminal B, 81% for HER2-enriched, 66% for basal-like, and 89% for normal-like. For tumor staging, the OS were 92% for IIA, 84% for IIB, 72% for IIIA, and 74% for IIIB.

**Table 5:** Overall cumulative survival probability at 60 months of Latin American women with breast cancer according to reproductive and hormonal characteristics, breast cancer family history, PAM50 subtype, and tumor clinical stage at diagnosis.

Reproductive, hormonal, and breast cancer history and characteristics of the tumor and treatment	Cumulative survival probability at 60 months (PS60)		Log-rank *p*-value
	PS60	95% CI	
Age range at menarche			
Up to 12 years	85.0	(80.9–88.2)	0.427
13 years and over	82.7	(78.8–86.0)	
Use of oral contraceptives			
Current or ever use	83.9	(80.4–86.9)	0.840
Never use	84.1	(79.6–87.8)	
At least one full pregnancy			
Yes	83.2	(80.3–85.8)	0.158
No	89.9	(80.0–95.0)	
Breastfeeding			
Yes	82.8	(79.7–85.5)	0.168
No^a^	87.4	(80.8–91.9)	
Menopause status and age			
Premenopausal	83.6	(78.8–87.4)	0.165
Up to 50 years	85.7	(81.4–89.1)	
51 years and over	79.4	(70.2–86.1)	
Self-reported previous benign breast diseases			
Yes	84.6	(81.0–87.6)	0.459
No	82.9	(78.5–86.5)	
First-degree family history of breast cancer			
Yes	88.2	(76.9–94.2)	0.353
No	83.5	(80.7–86.0)	
PAM50 intrinsic subtype			
Luminal A	92.6	(89.3–94.9)	<0.001
Luminal B	79.8	(73.1–85.0)	
HER2-enriched	80.8	(71.5–87.4)	
Basal-like	66.3	(57.4–73.7)	
Normal-like	88.8	(76.8–94.8)	
Tumor clinical stage at diagnosis			
IIA	92.3	(88.7–94.8)	<0.001
IIB	84.3	(79.2–88.2)	
IIIA	71.9	(64.3–78.1)	
IIIB	73.8	(62.3–82.2)	

a Includes never pregnant women.

**Figure 1:**
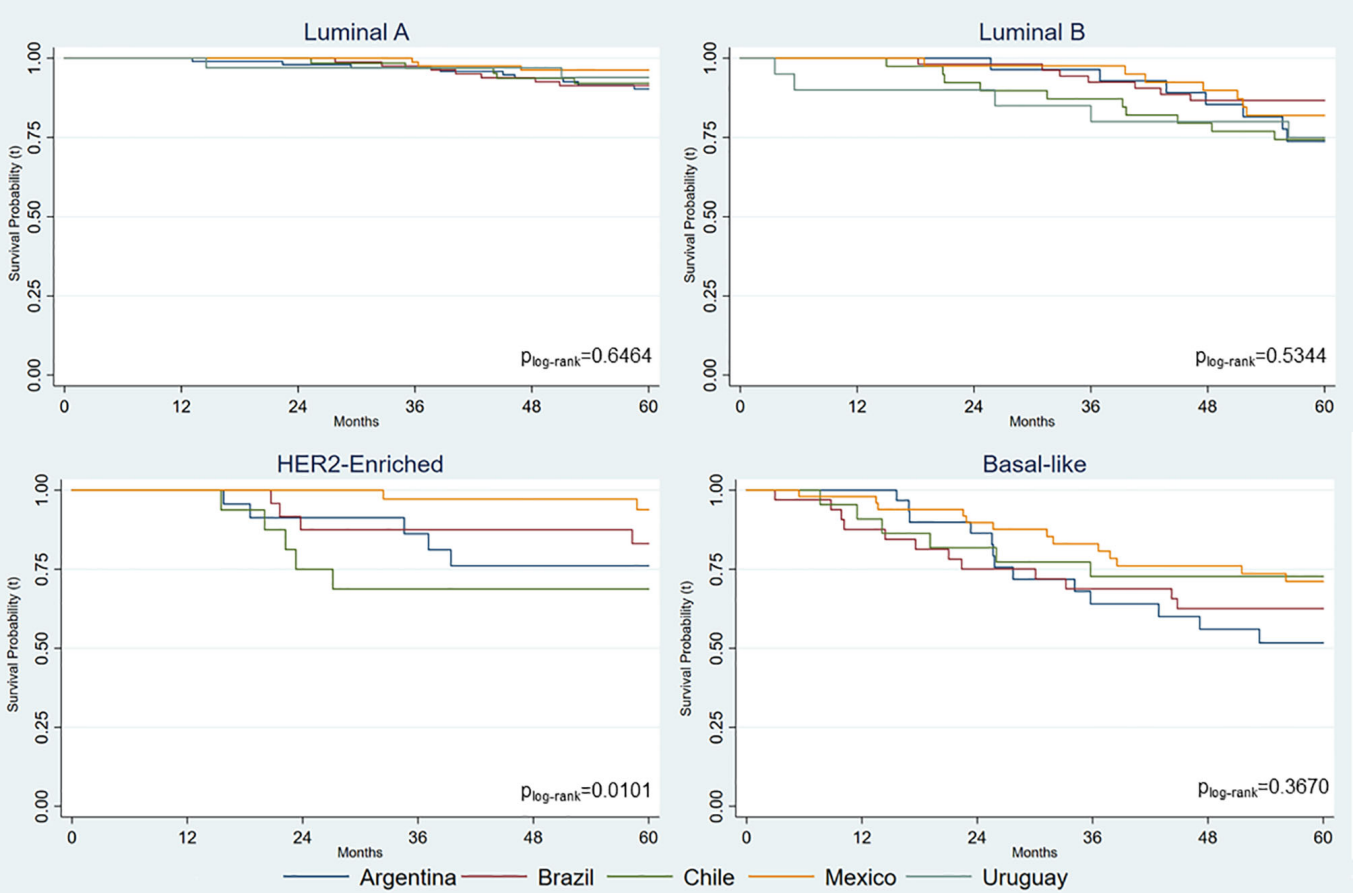
Overall cumulative survival probabilities for Latin American women with breast cancer by country of residence and PAM50 intrinsic subtype.

The OS by country of residence and PAM50 intrinsic subtypes indicated statistically significant differences (*p* = 0.010) only for HER2-enriched subtype (**Figure 1**). Mexico and Chile had the highest and lowest OS, respectively, for this subtype.

The multivariable-adjusted all-cause hazard ratios (HRs) for country of residence, age group, PAM50 intrinsic subtype, and tumor staging are shown on **Table 6**. The death hazards of women living in Argentina and Uruguay were more than twice as high as for Mexican women (referent). A similar difference in death hazards was seen between older (≥60 years) and younger (≤40 years) women. The HRs for luminal B and HER2-enriched subtypes were also twice as high as that for luminal A subtype. Death HR for basal-like subtype was 5.8 when compared with luminal A. As expected, death HRs for women diagnosed with clinical stages IIB, IIIA, and IIIB were higher than those diagnosed with stage IIA.

**Table 6:** Unadjusted and adjusted death hazard ratios of Latin American women with breast cancer by country of residence, age, education, PAM50 intrinsic subtypes, and tumor clinical stage at diagnosis.

	Unadjusted hazard ratio			Adjusted hazard ratio		
	HR	*p*-value	95% CI	HR	*p*-value	95% CI
Country of residence						
Mexico	1.00			1.00		
Argentina	1.69	0.047	(1.01–2.84)	2.27	0.003	(1.33–3.86)
Brazil	1.46	0.159	(0.86–2.48)	1.58	0.107	(0.91–2.74)
Chile	1.73	0.051	(1.00–2.99)	1.95	0.021	(1.11–3.44)
Uruguay	1.52	0.251	(0.74–3.10)	2.42	0.019	(1.16–5.04)
Age group						
Up to 40 years	1.00			1.00		
40 to 49 years	0.88	0.698	(0.46–1.67)	1.07	0.846	(0.56–2.04)
50 to 59 years	0.98	0.948	(0.53–1.80)	1.16	0.634	(0.63–2.15)
60 years and over	1.10	0.746	(0.61–2.01)	1.84	0.055	(0.99–3.43)
Education (years)	0.97	0.177	(0.94–1.01)	0.97	0.210	(0.93–1.02)
PAM50 intrinsic subtype						
Luminal A	1.00			1.00		
Luminal B	2.78	<0.001	(1.67–4.64)	2.43	0.001	(1.44–4.10)
HER2-enriched	2.72	0.001	(1.51–4.92)	2.52	0.003	(1.37–4.63)
Basal-like	5.76	<0.001	(3.54–9.35)	5.79	<0.001	(3.48–9.64)
Normal-like	1.52	0.359	(0.62–3.68)	1.32	0.543	(0.54–3.26)
Tumor clinical stage at diagnosis						
IIA	1.00			1.00		
IIB	2.11	0.004	(1.27–3.51)	1.91	0.015	(1.32–3.24)
IIIA	4.19	<0.001	(2.55–6.86)	3.54	<0.001	(2.10–5.98)
IIIB	3.92	<0.001	(2.17–7.10)	3.94	<0.001	(2.14–7.24)

Obs: the death hazard ratio for each variable is simultaneously and reciprocally adjusted for all other variables.

### General and Between-PAM50 Differences in Breast Cancer Risk Factors

Sociodemographic characteristics, lifestyle, reproductive and hormonal characteristics, family history of breast cancer, as well as tumor characteristics and treatment according to PAM50 intrinsic subtypes are shown in **Tables 7** and **8**. Luminal A and luminal B subtypes were more common in women ≥60 years old. HER2-enriched, basal-like, and normal-like were more common in younger women (50–59 years). The highest proportion of ever smokers was observed in luminal A and B subtypes. Differences in PAM50 subtypes by education level, alcohol use, physical activity, and BMI were not statistically significant (**Table 7**).

**Table 7:** Distribution of Latin American women with breast cancer by PAM50 subtypes according to sociodemographic and lifestyle factors.

Sociodemographic conditions and lifestyle	PAM50 intrinsic subtype										Total		*p*-value^*^
	Luminal A		Luminal B		HER2-enriched		Basal-like		Normal-like				
	*n*	%	*n*	%	*n*	%	*n*	%	*n*	%	*n*	%	
Total	370	42.9	185	21.4	110	12.7	140	16.2	58	6.7	863	100.0	n.a.
Country of residence													
Argentina	105	28.4	30	16.2	24	21.8	32	22.9	18	31.0	209	24.2	0.005
Brazil	82	22.2	53	28.6	24	21.8	33	23.6	11	19.0	203	23.5	
Chile	63	17.0	39	21.1	16	14.5	22	15.7	9	15.5	149	17.3	
Mexico	86	23.2	43	23.2	40	36.4	50	35.7	15	25.9	234	27.1	
Uruguay	34	9.2	20	10.8	6	5.5	3	2.1	5	8.6	68	7.9	
Age group													
Less than 40 years	31	8.4	25	13.5	9	8.2	21	15.0	6	10.3	92	10.7	<0.001
40 to 49 years	85	23.0	36	19.5	25	22.7	45	32.1	17	29.3	208	24.1	
50 to 59 years	104	28.1	54	29.2	47	42.7	52	37.1	26	44.8	283	32.8	
60 years and over	150	40.5	70	37.8	29	26.4	22	15.7	9	15.5	280	32.4	
Education													
0 to 8 years	189	51.1	86	46.5	60	54.5	70	50.0	31	53.4	436	50.5	0.823
9 to 24 years	177	47.8	94	50.8	50	45.5	69	49.3	27	46.6	417	48.3	
Unknown	4	1.1	5	2.7	0	0.0	1	0.7	0	0.0	10	1.2	
Alcohol use													
None	230	62.2	111	60.0	73	66.4	90	64.3	38	65.5	542	62.8	0.764
Any	134	36.2	70	37.8	33	30.0	49	35.0	20	34.5	306	35.5	
Unknown	6	1.6	4	2.2	4	3.6	1	0.7	0	0.0	15	1.7	
Tobacco use													
Never smoke	196	53.0	97	52.4	73	66.4	87	62.1	42	72.4	495	57.4	0.018
Smoker	64	17.3	26	14.1	16	14.5	14	10.0	6	10.3	126	14.6	
Ex-smoker	108	29.2	59	31.9	20	18.2	38	27.1	10	17.2	235	27.2	
Unknown	2	0.5	3	1.6	1	0.9	1	0.7	0	0.0	7	0.8	
Physical activity													
No	173	46.8	92	49.7	48	43.6	58	41.4	29	50.0	400	46.3	0.454
Yes	191	51.6	86	46.5	58	52.7	82	58.6	29	50.0	446	51.7	
Unknown	6	1.6	7	3.8	4	3.6	0	0.0	0	0.0	17	2.0	
Body mass index													
<25.0 kg/m^2^	104	28.1	44	23.8	31	28.2	33	23.6	14	24.1	226	26.2	0.641
25.0–29.9 kg/m^2^	128	34.6	64	34.6	41	37.3	50	35.7	21	36.2	304	35.2	
≥30.0 kg/m^2^	116	31.4	67	36.2	24	21.8	45	32.1	17	29.3	269	31.2	
Unknown	22	5.9	10	5.4	14	12.7	12	8.6	6	10.3	64	7.4	

^*^The p-value of the Chi-square test does not include the category unknown. n.a., Not applicable.

Women with stage IIA of breast cancer at diagnosis were more frequent in the luminal A (50.0%) and luminal B (36%) subtypes, while women with basal-like subtype were more frequently diagnosed at stage IIIA (36%). Differences in age range of menarche, use of oral contraceptives, at least one full pregnancy, breastfeeding, menopausal status and age, hormone replacement therapy, previous benign breast diseases, and first-degree family history of breast cancer were not statistically significant between PAM50 subtypes (**Table 8**).

**Table 8:** Distribution of Latin American women with breast cancer by PAM50 subtypes according to reproductive and hormonal characteristics, previous benign breast diseases, first-degree family history of breast cancer, and tumor clinical stage at diagnosis.

Reproductive, hormonal and breast cancer history, and characteristics of the tumor and treatment	PAM50 intrinsic subtype										Total		*p*-value^*^
	Luminal A		Luminal B		HER2-enriched		Basal-like		Normal				
	*n*	%	*n*	%	*n*	%	*n*	%	*n*	%	*n*	%	
Age range at menarche													
Up to 12 years	174	47.0	88	47.6	35	31.8	65	46.4	26	44.8	388	45.0	0.078
13 years and over	193	52.2	94	50.8	72	65.5	72	51.4	32	55.2	463	53.7	
Unknown	3	0.8	3	1.6	3	2.7	3	2.1	0	0.0	12	1.4	
Oral contraceptive use													
Ever use	227	61.4	116	62.7	57	51.8	89	63.6	32	55.2	521	60.4	0.354
Never use	141	38.1	67	36.2	50	45.5	51	36.4	26	44.8	335	38.8	
Unknown	2	0.5	2	1.1	3	2.7	0	0.0	0	0.0	7	0.8	
At least one full pregnancy													
Yes	335	90.5	160	86.5	101	91.8	132	94.3	53	91.4	781	90.5	0.316**
No	31	8.4	22	11.9	7	6.4	8	5.7	4	6.9	72	8.3	
Unknown	4	1.1	3	1.6	2	1.8	0	0.0	1	1.7	10	1.2	
Breastfeeding													
Yes	301	81.4	141	76.2	82	74.5	117	83.6	49	84.5	690	80.0	0.125
No^a^	59	15.9	40	21.6	26	23.6	21	15.0	7	12.1	153	17.7	
Unknown	10	2.7	4	2.2	2	1.8	2	1.4	2	3.4	20	2.3	
Menopause status and age													
Premenopausal	135	36.5	57	30.8	33	30.0	52	37.1	24	41.4	301	34.9	0.099
Up to 50 years	147	39.7	88	47.6	43	39.1	51	36.4	21	36.2	350	40.6	
51 years and over	53	14.3	16	8.6	20	18.2	13	9.3	5	8.6	107	12.4	
Unknown	35	9.5	24	13.0	14	12.7	24	17.1	8	13.8	105	12.2	
Hormone replacement therapy													
Yes	37	10.0	27	14.6	14	12.7	22	15.7	4	6.9	104	12.1	
No	330	89.2	153	82.7	94	85.5	117	83.6	54	93.1	748	86.7	0.188^**^
Unknown	3	0.8	5	2.7	2	1.8	1	0.7	0	0.0	11	1.3	
Self-reported previous benign breast diseases													
Yes	223	60.3	112	60.5	57	51.8	73	52.1	35	60.3	500	57.9	0.277
No	147	39.7	73	39.5	53	48.2	67	47.9	23	39.7	363	42.1	
First-degree family history of breast cancer													
Yes	31	8.4	16	8.6	6	5.5	9	6.4	1	1.7	63	7.3	0.352
No	339	91.6	169	91.4	104	94.5	131	93.6	57	98.3	800	92.7	
Tumor clinical stage at diagnosis													
IIA	185	50.0	66	35.7	33	30.0	30	21.4	17	29.3	331	38.4	<0.001
IIB	116	31.4	58	31.4	34	30.9	47	33.6	19	32.8	274	31.7	
IIIA	35	9.5	43	23.2	31	28.2	50	35.7	15	25.9	174	20.2	
IIIB	34	9.2	18	9.7	12	10.9	13	9.3	7	12.1	84	9.7	

^*^The p-value of Chi-square test does not include the category unknown. ^**^The p-value of Fisher’s test does not include the category Unknown. ^a^Includes never pregnant women.

## Discussion

This paper reports the first epidemiological and molecular analysis of a study carried out by the LACRN in collaboration with the National Cancer Institute/USA. The general objective of this network is to strengthen collaborative research efforts among the participating countries, advance translational cancer research, and serve as a basis for planning new actions to reduce the global cancer burden, focusing on breast cancer as a priority (14).

With regard to the characteristics of breast cancer included in our study, distribution by age in Latin American women is in line with what has been observed in previous studies, in which the majority of cases have been aged 50 years and older. Age is one of the most important risk factors for breast cancer. It is projected that the number of women aged 60 years and older will double by 2030, with age-unadjusted breast cancer incidence probably following this tendency (12). Uruguay and Argentina had the highest breast cancer rates in Latin American women in 2013 (12). As these rates were age-adjusted, other-than-age demographic, behavioral and reproductive factors may be responsible for the high rates in these countries. In developed countries, the breast cancer incidence rates are high while breast cancer mortality rates are much lower (1).

Almost one-half of women in our study had menarche at age ≤12 years and one in five experienced menopause onset at ages ≥50 years. Last century’s changes in sexual maturation and reproductive patterns have led to an increased exposure to estrogen and progesterone levels, contributing to increased risk of breast cancer (17). Indeed, breast cancer risk increases by 5% for each younger year at menarche and ≈3% for each older year in menopause onset (18). Younger age at menopause has directly been found to be associated with luminal-like tumors but not with HER2-enriched or basal-like subtypes (19).

The majority of Latin American women in our study had at least one full-term pregnancy and breastfed their children, both of which are protective against breast cancer (20).

With regard to the main objective of our study, compared with the death hazard ratio (HR) for luminal A, the death hazards for luminal B and HER-2 enriched subtypes were more than twice as high. The hazard for the basal-like subtype was about 6 times higher than that for luminal A confirming the latter as the least aggressive subtype. Worst cumulative survival probabilities were found for HER2-enriched and basal-like subtypes in a previous study (21).

The overall cumulative survival probability curves by molecular subtypes of breast cancer by country in our study reflected a combination of factors. In addition to molecular subtype, OS also reflected tumor staging and age at diagnosis and clinical and socioeconomic conditions. While socioeconomic status can influence access to care (including access to early diagnosis and to treatment with specific drugs), it may also influence epigenetic changes that influence molecular subtypes (22–25).

Women were treated according to the institutions’ standard protocols, in which the access to some expensive drugs depends on the healthcare organization in each country and on individual health insurance. These are likely reasons why some breast cancer patients in Chile and Argentina did not receive specific drugs that are known to improve the prognosis (26). Access to diagnosis and treatment makes it possible for women with breast cancer to be diagnosed at an earlier stage, which would improve their survival. Identification of molecular profiles at diagnosis is particularly important because they result in the implementation of more efficacious treatment protocols. This likely impacted the OS in these countries, especially in women with the HER2-enriched subtype of breast cancer.

Life expectancy at birth is the integrated survivorship of the population across all ages, and it is used as a proxy of population health (27). Life expectancy has significant effect on *per capita* income as well as its growth. Education is also positively associated with the *per capita* income. High prevalence of the disease burden in working age group implies a significant loss of human capital which impairs its contribution to economic production (28). Other studies demonstrated that GPD *per capita* and education level explain 72.6% to 82.6% differences in life expectancy at birth (29). In 2010, Chile and Uruguay had the highest life expectancy of women at birth, and, in 2013, Chile and Argentina had the highest GPD *per capita* and Human Development Index (**Supplementary Figure S1**). Yet, in our study, women with breast cancer in Mexico and Brazil showed higher overall cumulative survival than Argentina, Chile, and Uruguay. One possible factor, not explored here, is differences in genetic susceptibility in these countries. Fejerman et al. (30) evaluated the association between genetic ancestry and survival after breast cancer diagnosis among US Latin American women and concluded that genetic factors and/or unmeasured differences in treatment or access to care should be explored to better understand disparities in breast cancer outcome (30). To evaluate these factors, we are developing new molecular studies related to ancestry in these women. Other important prognostic factors that should be explored in future studies are the organization of the health system and women’s behavior related to preventive practices as well as their access to healthcare.

A major strength of the study was its multicenter feature and careful development of the questionnaire and the implementation of standardized protocols in five different countries. Although this study was conducted over a large and diverse geographic area, results are likely generalizable to women who receive their breast cancer care in similar healthcare settings. All protocols for the tumor molecular characterization were standardized in order to establish the PAM50 or set of 50 “intrinsic” genes that allowed the classification of breast cancer subtypes to be hypothesized as prognostic factors.

Among the study limitations, there was the loss of epidemiological, clinical, and molecular information. Identifying a series of 863 breast cancer cases across five major geographic areas may influence precision of our findings, especially with regard to between-country comparisons. Another potential limitation includes the differences in health systems, social determinants of health, and inequity in access to healthcare or supply of appropriated therapies. Notwithstanding this limitation, we believe that the evidence generated in this study is of value for all countries and may help in improving identification and treatment of breast cancer.

In conclusion, the overall cumulative survival in our study was associated with molecular subtype of the tumor, age, and tumor stage at diagnosis. The latter can reflect contextual variables that, in turn, likely influence access to early diagnosis and treatment of the breast cancer. While the latter is known to be influenced by access to care, including cancer screening, timely diagnosis, and treatment, as well as access to more effective treatment protocols (22–24), it may also influence epigenetic changes that, potentially, can impact molecular subtypes.

## United States-Latin American Cancer Research Network (US-LACRN)

Juan Abarca (Hospital Barros Luco Trudeau, Santiago, Chile), Pamela Acevedo (Hospital San José, Santiago, Chile), Graciela Acosta (Hospital Municipal de Oncología María Curie, Buenos Aires, Argentina), Ana Acosta (Universidad de Sonora, Hermosillo, Mexico), Gabriela Acosta Haab (Hospital Municipal de Oncología María Curie, Buenos Aires, Argentina), Keyla Teresa Acosta-Torres (Hospital General Zona 2, IMSS, Hermosillo, México), Marta Aghazarian (Instituto Nacional de Cáncer, Montevideo, Uruguay), Carola Aguayo (Instituto de Salud Pública, Santiago, Chile), Gustavo Alarcon-Lopez (Hospital Integral de la Mujer en Estado de Sonora, Hermosillo, México), Viviane Andrade (Hospital de Câncer de Barretos, Barretos, Brazil), Wenceslao Angeles-Bueno (Hospital de Especialidades, CMNO-IMSS, Guadalajara, Mexico), Roberto Arai (Instituto de Câncer de São Paulo, São Paulo, Brazil), Priscila Elvira Arambula-Barreras (Universidad de Sonora, Hermosillo, Mexico), Maria Isabel Arámburo-Rubio (Hospital General Zona 2, IMSS, Hermosillo, México), Estrellita Araus (Hospital Barros Luco Trudeau, Santiago, Chile), Gonzalo Ardao (Hospital Central de las Fuerzas Armadas, Montevideo, Uruguay), Lilia A. Arellano-Jimenez (Universidad de Guadalajara, Guadalajara, Mexico), Felipe Argandoña (Hospital San Borja Arriarán, Santiago, Chile), Claudia Arias (Hospital Municipal de Oncología María Curie, Buenos Aires, Argentina), Ricardo Armisen (Universidad de Chile, Santiago, Chile), Mauricio Aspee (Hospital Luis Tisne, Santiago, Chile), Rodrigo Assar (Universidad de Chile, Santiago, Chile), Itzel Reneé Astiazarán-Rascón (Universidad de Sonora, Hermosillo, Mexico), Sebastian Astorga (Hospital San Borja Arriarán, Santiago, Chile), Maxwell Avilés-Rodríguez (Centro Estatal de Oncologia, Hermosillo, México), Antônio Bailão Junior (Hospital de Câncer de Barretos, Barretos, Brazil), Adolfo E. Barragan-Curiel (OPD Hospital Civil de Guadalajara, Universidad de Guadalajara, Guadalajara, Mexico), Adelfo Barragan-Ruiz (Hospital de Gineco-Obstricia, CMNO-IMSS, Guadalajara, Mexico), Julia Bernachin (Hospital de Clínicas Manuel Quintela, Universidad de la República, Montevideo, Uruguay), Wilfrido Bernal-Herrera (Centro Estatal de Oncologia, Hermosillo, México), Renata Binato (Coordination of Prevention and Surveillance, Instituto Nacional de Câncer, Rio de Janeiro, Brazil), Sarah Brnich (Fundación Instituto Leloir, CONICET, Buenos Aires, Argentina), Claudio Bustamante (Hospital San José, Santiago, Chile), Miguel Angel Bustamante (Hospital San Borja Arriarán, Santiago, Chile), Julio Bustos-Gomez (OPD Hospital Civil de Guadalajara, Universidad de Guadalajara, Guadalajara, Mexico), Felipe de J. Bustos-Rodriguez (OPD Hospital Civil de Guadalajara, Universidad de Guadalajara, Guadalajara, Mexico), Janett Caballero-Jasso (Hospital General Zona 14, IMSS, Hermosillo, México), Angie Calfuman (Hospital Luis Tisne, Santiago, Chile), Antonio Hugo José Froes Marques Campos (AC Camargo Cancer Center, São Paulo, Brazil), Mónica Campos (Hospital San Borja Arriarán, Santiago, Chile), Soledad Cano (Instituto Nacional del Cáncer, Santiago, Chile), Juan C. Canton-Romero (Hospital de Gineco-Obstricia, CMNO-IMSS, Guadalajara, Mexico), Paulina Carmona (Grupo Oncológico Cooperativo Chileno de Investigación, Santiago, Chile), Fernando Carrizo (Instituto de Oncología Angel Roffo, Buenos Aires, Argentina), André Lopes Carvalho (Hospital de Câncer de Barretos, Barretos, Brazil), Erika Carvallo (Hospital San Borja Arriarán, Santiago, Chile), Julio Carzoglio (Instituto Nacional del Cáncer, Santiago, Chile), Monica Casalnuovo (Hospital Municipal de Oncología María Curie, Buenos Aires, Argentina), Benedicta Caserta (Centro Hospitalario Pereira Rossell, Montevideo, Uruguay), Alvaro Castillo (Hospital San José, Santiago, Chile), César Castillo (Hospital Barros Luco Trudeau, Santiago, Chile), Juan M. Castro-Cervantes (Hospital de Especialidades, CMNO-IMSS, Guadalajara, Mexico), Yascara Cerda (Grupo Oncológico Cooperativo Chileno de Investigación, Santiago, Chile), Roger Chammas (Instituto de Câncer de São Paulo, São Paulo, Brazil), Mario Alberto Chavez-Zamudio (Hospital General No 1, IMSS, Obregon, México), Loreto Chia (Hospital San José, Santiago, Chile), Daniela Chirico (Fundación Instituto Leloir, CONICET, Buenos Aires, Argentina), Esther Cisneros-Quirarter (Universidad de Guadalajara, Guadalajara, Mexico), Minor Raul Cordero-Bautista (Hospital Luis Tisne, Santiago, Chile), Valeria Cornejo (Hospital San Borja Arriarán, Santiago, Chile), Baldemar Corral-Villegas (Centro Estatal de Oncologia, Hermosillo, México), Andrés Cortés (Grupo Oncológico Cooperativo Chileno de Investigación, Santiago, Chile), German Salvador Cortez-Zamorano (Universidad de Sonora, Hermosillo, Mexico), Alejandro Corvalan (Grupo Oncológico Cooperativo Chileno de Investigación, Santiago, Chile), Adolfo Cruz (Hospital Barros Luco Trudeau, Santiago, Chile), Alba d’Aurora (Hospital San Borja Arriarán, Santiago, Chile), Sandra De la Fuente (Universidad de Chile, Santiago, Chile), Soledad De la Peña (Centro Hospitalario Pereira Rossell, Montevideo, Uruguay), Roberto de Leon-Caballero (Hospital General del Estado de Sonora, Hermosillo, México), César Del Castillo (Hospital San Borja Arriarán, Santiago, Chile), Azucena Del-Toro-Valero (Universidad de Guadalajara, Guadalajara, Mexico), Mirtha Di Pretoro (Instituto de Oncología Angel Roffo, Buenos Aires, Argentina), Andrea Digonzelli (Hospital Regional de Agudos Eva Perón, Buenos Aires, Argentina), Jose El Ters (Instituto Nacional de Cáncer, Montevideo, Uruguay), Paula Escobar (Hospital Luis Tisne, Santiago, Chile), Marcela Estolaza (Hospital Luis Tisne, Santiago, Chile), Adriane Feijo Evangelista (Hospital de Câncer de Barretos, Barretos, Brazil), Marcelo Fanelli (AC Camargo Cancer Center, São Paulo, Brazil), Paulo Farias (Coordination of Prevention and Surveillance, Instituto Nacional de Câncer, Rio de Janeiro, Brazil), Diego Flaks (Hospital Municipal de Oncología María Curie, Buenos Aires, Argentina), Edgar G. Flores-Ayala (Instituto Jalisciense de Cancerologia, Guadalajara, Mexico), Maria R. Flores-Marquez (Hospital de Especialidades, CMNO-IMSS, Guadalajara, Mexico), David Franco-Hughes (Universidad de Sonora, Hermosillo, Mexico), Karina Franco-Topete (OPD Hospital Civil de Guadalajara, Universidad de Guadalajara, Guadalajara, Mexico), Cristobal Fresno (Universidad Católica de Córdoba, Centro de Investigaciones en Bioquímica Clínica e Inmunología-CONICET, Córdoba, Argentina), Carolina Gabay (Instituto de Oncología Angel Roffo, Buenos Aires, Argentina), Mario Gallegos (Hospital San Borja Arriarán, Santiago, Chile), Jorge Gamboa (Hospital San Borja Arriarán, Santiago, Chile), Daiana Ganiewich (Fundación Instituto Leloir, CONICET, Buenos Aires, Argentina), Carlos Garbovesky (Hospital Municipal de Oncología María Curie, Buenos Aires, Argentina), Ricardo Garcia-Gaeta (Universidad de Guadalajara, Guadalajara, Mexico), Alma C. Garcia-Martinez (Universidad de Guadalajara, Guadalajara, Mexico), Rubén Alejandro García-Munguía (Hospital de Ginecopediatria, Hermosillo, México), Adriana Garibay-Escobar (Universidad de Sonora, Hermosillo, Mexico), Liliana Gimenez (Instituto de Oncología Angel Roffo, Buenos Aires, Argentina), Mariana M. Gomez-Del Toro (Universidad de Guadalajara, Guadalajara, Mexico), Germán González (Universidad Católica de Córdoba, Centro de Investigaciones en Bioquímica Clínica e Inmunología-CONICET, Córdoba, Argentina), César Osbaldo González-Mondaca (Hospital General Zona 2, IMSS, Hermosillo, México), Leivy P. Gonzalez-Ramirez (Universidad de Guadalajara, Guadalajara, Mexico), Beatriz Gonzalez-Ulloa (Hospital de Especialidades, CMNO-IMSS, Guadalajara, Mexico), Susana Gorostidy (Instituto de Oncología Angel Roffo, Buenos Aires, Argentina), Gonzalo Greif (Institut Pasteur de Montevideo, Montevideo, Uruguay), Alfonso G.Guevara-Torres (Centro Estatal de Oncologia, Hermosillo, México), Lorena Gutierrez (Hospital San Borja Arriarán, Santiago, Chile), Adrián Hannois (Hospital Regional de Agudos Eva Perón, Buenos Aires, Argentina), Andrew Hart (Universidad de Chile, Santiago, Chile), Steffen Härtel (Universidad de Chile, Santiago, Chile), Marcos Henriquez (Hospital Barros Luco Trudeau, Santiago, Chile), Miriam E Hernandez-Franco (Universidad de Guadalajara, Guadalajara, Mexico), Rafael Hernandez-Guevara (Hospital General No 1, IMSS, Obregon, México), Manuel I Herrera-Miramontes (Universidad de Guadalajara, Guadalajara, Mexico), Graciela Horton (Hospital Municipal de Oncología María Curie, Buenos Aires, Argentina), Gladys Ibañez (Hospital San José, Santiago, Chile), Martín Ipiña (Instituto de Oncología Angel Roffo, Buenos Aires, Argentina), Lilian Jara (Universidad de Chile, Santiago, Chile), Raul Jara (Hospital Luis Tisne, Santiago, Chile), Maria Luisa Jaramillo (Hospital San Borja Arriarán, Santiago, Chile), Beatriz Jardim (Coordination of Prevention and Surveillance, Instituto Nacional de Câncer, Rio de Janeiro, Brazil), Maria Eugenia Jimenez (Hospital Barros Luco Trudeau, Santiago, Chile), Victor M. Jimenez-Moreno (Hospital de Gineco-Obstricia, CMNO-IMSS, Guadalajara, Mexico), Hugo Ju (Grupo Oncológico Cooperativo Chileno de Investigación, Santiago, Chile), Nazareth Juárez Rusjan (Instituto de Oncología Angel Roffo, Buenos Aires, Argentina), Karen Juneman (Hospital Luis Tisne, Santiago, Chile), Ligia Maria Kerr (Hospital de Câncer de Barretos, Barretos, Brazil), Alejandra Krupelis (Hospital Municipal de Oncología María Curie, Buenos Aires, Argentina), Guillermo Laviña (Hospital de Clínicas Manuel Quintela, Universidad de la República, Montevideo, Uruguay), Fernando Lavista (Hospital Central de las Fuerzas Armadas, Montevideo, Uruguay), Irma Leticia León-Duarte (Hospital General del Estado de Sonora, Hermosillo, México), Alberto Lescano (Hospital Municipal de Oncología María Curie, Buenos Aires, Argentina), Verónica Lezano (Grupo Oncológico Cooperativo Chileno de Investigación, Santiago, Chile), Rossana Mendoza Lopez (Instituto de Câncer de São Paulo, São Paulo, Brazil), Jose Guillermo López-Cervantes (Universidad de Sonora, Hermosillo, Mexico), Miguel Enrique Lopez-Muñoz (Universidad de Sonora, Hermosillo, Mexico), Francisca Lucena (Coordination of Prevention and Surveillance, Instituto Nacional de Câncer, Rio de Janeiro, Brazil), Alejandra Luque (Hospital Central de las Fuerzas Armadas, Montevideo, Uruguay), Alejandro Maass (Universidad de Chile, Santiago, Chile), Maria do Socorro Maciel (AC Camargo Cancer Center, São Paulo, Brazil), Silvina Maldonado (Hospital Regional de Agudos Eva Perón, Buenos Aires, Argentina), Flavia Rotea Mangone (Instituto de Câncer de São Paulo, São Paulo, Brazil), Jorge Mansilla (Universidad de Chile, Santiago, Chile), Katherine Marcelain (Universidad de Chile, Santiago, Chile), Carolina Mariani (Grupo Oncológico Cooperativo Chileno de Investigación, Santiago, Chile), Marcia Maria Chiquitelli Marques (Hospital de Câncer de Barretos, Barretos, Brazil), Reyna J Martinez-Arriaga (Universidad de Guadalajara, Guadalajara, Mexico), Hector R Martinez-Ramirez (Hospital de Especialidades, CMNO-IMSS, Guadalajara, Mexico), Marcela Martins (Instituto de Câncer de São Paulo, São Paulo, Brazil), Alma G Maya-Gonzalez (Universidad de Guadalajara, Guadalajara, Mexico), Mariana Menini (Hospital Central de las Fuerzas Armadas, Montevideo, Uruguay), Soledad Milans (Hospital de Clínicas Manuel Quintela, Universidad de la República, Montevideo, Uruguay), Soledad Montes (Instituto Nacional del Cáncer, Santiago, Chile), Ana Verónica Morales-Hernández (Universidad de Sonora, Hermosillo, Mexico), Carla Morong (Hospital San Borja Arriarán, Santiago, Chile), Eduardo Mussetti (Centro Hospitalario Pereira Rossell, Montevideo, Uruguay), Luis J Najar-Acosta (Universidad de Guadalajara, Guadalajara, Mexico), Elisa Napolitano e Ferreira (AC Camargo Cancer Center, São Paulo, Brazil), Nancy E Navarro-Ruiz (Universidad de Guadalajara, Guadalajara, Mexico), Cristina Noblía (Instituto de Oncología Angel Roffo, Buenos Aires, Argentina), João Soares Nunes (Hospital de Câncer de Barretos, Barretos, Brazil), Fabiola Núñez (Hospital Luis Tisne, Santiago, Chile), Nilton Onari (Hospital de Câncer de Barretos, Barretos, Brazil), Emma M Oropeza-De Anda (Universidad de Guadalajara, Guadalajara, Mexico), David Ortega-Tirado (Universidad de Sonora, Hermosillo, Mexico), Miguel Angel Ortiz-Martinez (Hospital General No 1, IMSS, Obregon, México), Cynthia Aparecida Bueno de Toledo Osório (AC Camargo Cancer Center, São Paulo, Brazil), Carlos Eduardo Paiva (Hospital de Câncer de Barretos, Barretos, Brazil), Paulina Peñaloza (Hospital Luis Tisne, Santiago, Chile), Miguel Peredo-Navarro (Hospital de Especialidades, CMNO-IMSS, Guadalajara, Mexico), David Pereira (Instituto de Oncología Angel Roffo, Buenos Aires, Argentina), Laura Perez-Michel (Hospital General No 1, IMSS, Obregon, México), Francisca Pino (Hospital Barros Luco Trudeau, Santiago, Chile), Tania Pino (Hospital San José, Santiago, Chile), Natalia Pinto (Hospital Luis Tisne, Santiago, Chile), Jessica Pizarro (Hospital Barros Luco Trudeau, Santiago, Chile), Jael Quintero (Universidad de Sonora, Hermosillo, Mexico), Antonio Quintero-Ramos (Universidad de Guadalajara, Guadalajara, Mexico), Enrique Ramirez (Hospital Municipal de Oncología María Curie, Buenos Aires, Argentina), Gladys E Ramirez-Rosales (Universidad de Guadalajara, Guadalajara, Mexico), Claudia Ramis (Hospital San José, Santiago, Chile), Maritza Ramos-Ramirez (Universidad de Guadalajara, Guadalajara, Mexico), Adela Rascon-Alcantar (Hospital de Ginecopediatria, Hermosillo, México), Francois Richard (Grupo Oncológico Cooperativo Chileno de Investigación, Santiago, Chile), Omar Rios-Méndez (Hospital General del Estado de Sonora, Hermosillo, México), ErnestoRivera-Claisse (Centro Estatal de Oncologia, Hermosillo, México), Ramón E.Robles-Zepeda (Universidad de Sonora, Hermosillo, Mexico), Iara Santana Rocha (Hospital de Câncer de Barretos, Barretos, Brazil), Natalia Rodriguez (Hospital Barros Luco Trudeau, Santiago, Chile), Vilma Rodriguez (Hospital Barros Luco Trudeau, Santiago, Chile), Maria Teresa Rodriguez (Hospital San José, Santiago, Chile), Diego Rodriguez-Gonzalez (Universidad de Guadalajara, Guadalajara, Mexico), Rosemeire A Roela (Instituto de Câncer de São Paulo, São Paulo, Brazil), Ana M. Romero-Gomez (Universidad de Guadalajara, Guadalajara, Mexico), Ana M. Rosales-Sandoval (Hospital de Especialidades, CMNO-IMSS, Guadalajara, Mexico), Lidia A. Rubio-Chavez (Universidad de Guadalajara, Guadalajara, Mexico), Omar V. Rubio-Plascencia (Universidad de Guadalajara, Guadalajara, Mexico), Florencia Russo (Hospital Regional de Agudos Eva Perón, Buenos Aires, Argentina), Gaciela Sabini (Programa Nacional para el Control del Cancer, Montevideo, Uruguay), Isabel Saffie (Hospital Luis Tisne, Santiago, Chile), Brenda Samaniego (Universidad de Sonora, Hermosillo, Mexico), Benito Sanchez-Llamas (Hospital de Gineco-Obstricia, CMNO-IMSS, Guadalajara, Mexico), Verónica Sanchotena (Hospital Municipal de Oncología María Curie, Buenos Aires, Argentina), Daniel Sat-Muñoz (Hospital de Gineco-Obstricia, CMNO-IMSS, Guadalajara, Mexico), Mariana Savignano (Instituto de Oncología Angel Roffo, Buenos Aires, Argentina), Cristovam Scapulatempo Neto (Hospital de Câncer de Barretos, Barretos, Brazil), Max Mano Senna (Instituto de Câncer de São Paulo, São Paulo, Brazil), Carolina Silva (Hospital San Borja Arriarán, Santiago, Chile), Jaime Silvera (Centro Hospitalario Pereira Rossell, Montevideo, Uruguay), Isabele Small (Coordination of Prevention and Surveillance, Instituto Nacional de Câncer, Rio de Janeiro, Brazil), Fernando Soares (Coordination of Prevention and Surveillance, Instituto Nacional de Câncer, Rio de Janeiro, Brazil), Iberê Soares (Instituto de Câncer de São Paulo, São Paulo, Brazil), Silvana Soares dos Santos (AC Camargo Cancer Center, São Paulo, Brazil), Evandro Sobrosa de Mello (Instituto de Câncer de São Paulo, São Paulo, Brazil), José Antonio Sola (Instituto Nacional del Cáncer, Santiago, Chile), Irene Sorín (Hospital Regional de Agudos Eva Perón, Buenos Aires, Argentina), Alejandra Sosa (Programa Nacional para el Control del Cancer, Montevideo, Uruguay), Claudio Sosa (Centro Hospitalario Pereira Rossell, Montevideo, Uruguay), Cristiano de Pádua Souza (Hospital de Câncer de Barretos, Barretos, Brazil), Lucía Spangenberg (Institut Pasteur de Montevideo, Montevideo, Uruguay), Gustavo Steffanof (Coordination of Prevention and Surveillance, Instituto Nacional de Câncer, Rio de Janeiro, Brazil), Florencia Straminsky (Fundación Instituto Leloir, CONICET, Buenos Aires, Argentina), Mónica Tapia (Hospital Luis Tisne, Santiago, Chile), Raziel O. Tapia-Llanos (Universidad de Guadalajara, Guadalajara, Mexico), Geronimo M. Tavares-Macias (Hospital de Especialidades, CMNO-IMSS, Guadalajara, Mexico), Veronica Terzieff (Centro Hospitalario Pereira Rossell, Montevideo, Uruguay), Vicente Teti (Hospital Municipal de Oncología María Curie, Buenos Aires, Argentina), Javier Tognarelli (Instituto de Salud Pública, Santiago, Chile), Verónica Toledo (Hospital Luis Tisne, Santiago, Chile), Paulina Toro (Hospital Luis Tisne, Santiago, Chile), Roberto Torres (Instituto Nacional del Cáncer, Santiago, Chile), Mariana Torres-Palomares (Universidad de Guadalajara, Guadalajara, Mexico), Alejandra Trinchero (Hospital Regional de Agudos Eva Perón, Buenos Aires, Argentina), Rogelio Troyo-San Roman (Universidad de Guadalajara, Guadalajara, Mexico), Hernan Urbano (Hospital Barros Luco Trudeau, Santiago, Chile), Nicolas Vacca (Programa Nacional para el Control del Cancer, Montevideo, Uruguay), María Lourdes Valencia-Peña (Universidad de Sonora, Hermosillo, Mexico), Jaime Vazquez-Nares (Instituto Jalisciense de Cancerologia, Guadalajara, Mexico), Ezequiel Velez-Gomez (OPD Hospital Civil de Guadalajara, Universidad de Guadalajara, Guadalajara, Mexico), Laura N. Venegas-Godinez (Universidad de Guadalajara, Guadalajara, Mexico), Ricardo Verdugo (Universidad de Chile, Santiago, Chile), René Aloisio da Costa Vieira (Hospital de Câncer de Barretos, Barretos, Brazil), Manuel Isaac Villegas-Gómez (Universidad de Guadalajara, Guadalajara, Mexico), Silvia Vornetti (Hospital Municipal de Oncología María Curie, Buenos Aires, Argentina), Anapaula H. U. Watanabe (Hospital de Câncer de Barretos, Barretos, Brazil), Carlos Zamorano (Hospital Barros Luco Trudeau, Santiago, Chile), Luis Zapata (Hospital Barros Luco Trudeau, Santiago, Chile), Zdenka Zlatar (Grupo Oncológico Cooperativo Chileno de Investigación, Santiago, Chile).

## Data Availability Statement

The raw data supporting the conclusions of this article will be made available by the authors, without undue reservation.

## Ethics Statement

The studies involving human participants were reviewed and approved by the National Cancer Institute ethics committee. The patients/participants provided their written informed consent to participate in this study.

## Author Contributions

Conceptualization, design, investigation, supervision, data collection, data curation, statistical analysis, writing, reviewing, and editing: LA, SCo, MV, OV, LC-S, MSo, and RB. Conceptualization, supervision, investigation and data collection, funding acquisition, reviewing, and editing: EAb, NA, AD-N, AL, BM, OP, CV, MB, and JF. Conceptualization, interpretation, writing, reviewing, and editing: BJo and MSz. Investigation and data collection: EAl, IA, AB, NC, DC, MCa, SCa, ACa, MCe, ACo, SCr, AT-A, RD-C, LD, EF, JF, WF, RF-T, FG, JG, LG-R, MG, SG-R, BJa, AL-V, DL, SM, AM-M, GM-V, CM, MN, AO-V, RRe, JR, RRo, CR, ES-G, LS, JS, AS-G, SV, LZ, and United States-Latin American Cancer Research Network (US-LACRN). All authors listed have made a substantial, direct, and intellectual contribution to the work and approved it for publication.

## Funding

This study was partially supported by the Center for Global Health at the United States - National Cancer Institute at the National Institutes of Health (contract award # HHSN2612010000871/NO2-PC-2010-00087) and by the official agencies of each country.

## Conflict of Interest

The authors declare that the research was conducted in the absence of any commercial or financial relationships that could be construed as a potential conflict of interest.

## Publisher’s Note

All claims expressed in this article are solely those of the authors and do not necessarily represent those of their affiliated organizations, or those of the publisher, the editors and the reviewers. Any product that may be evaluated in this article, or claim that may be made by its manufacturer, is not guaranteed or endorsed by the publisher.
