# Supplementary material for: Socioeconomic, Clinical, and Molecular Features of Breast Cancer Influence Overall Survival of Latin American Women
Source: Front Oncol. 2022 Mar 8;12:845527. doi: 10.3389/fonc.2022.845527 (PMC9071365; doi:10.3389/fonc.2022.845527)
Supplement: Supplementary file 1 [file DataSheet_1.zip › Supplementary Material/Supplementary Material 1 - 3.pdf]

Socioeconomic, clinical and molecular features of breast cancer influence overall survival of Latin American women

SUPPLEMENTAL MATERIAL 1

Figure S1: Population size, life expectancy of women at birth, GDP per capita, HDI, estimated number of deaths, crude rate and age-standardized rate (World) for breast cancer by Latin American countries, in 2013

Countries	Estimated Population size (2013)[1]	Life expectancy of women at birth (years) (2010)[2]	GDP per capita (US$) 2013[3]	HDI (2013)[4]	Estimated number of deaths, breast cancer, in 2013*[5]	Crude rate of Breast cancer 2013*[5]	ASR of Breast cancer 2013 (World)*[5]
**Argentina**	41,446,246	78.6	20,131.68	0.835	5,632	26.0	17.9
**Brazil**	200,361,925	77.7	15,588.73	0.753	14,204	13.9	11.8
**Chile**	17,619,708	81.3	22,439.28	0.826	1,389	15.6	10.5
**Mexico**	122,332,399	78.0	17,373.90	0.756	6,548	10.4	9.8
**Uruguay**	3,407,062	80.0	19,130.04	0.800	632	36.0	18.9

Notes: *per 100.000 women

Sources:[1]https://www.worldometers.info/world-population/world-population-countries.phps, [2]https://apps.who.int/gho/data/node.main.688?lang=en, [3]https://datos.bancomundial.org/indicator/NY.GDP.PCAP.PP.CD?end=2016&locations=CL&start=2010, [4]https://datosmacro.expansion.com/idh?anio=2013 and [5]https://www-dep.iarc.fr/WHOdb/WHOdb.htm

SUPPLEMENTAL MATERIAL 2: Figure S2: Schema for LACRN Breast Cancer Study

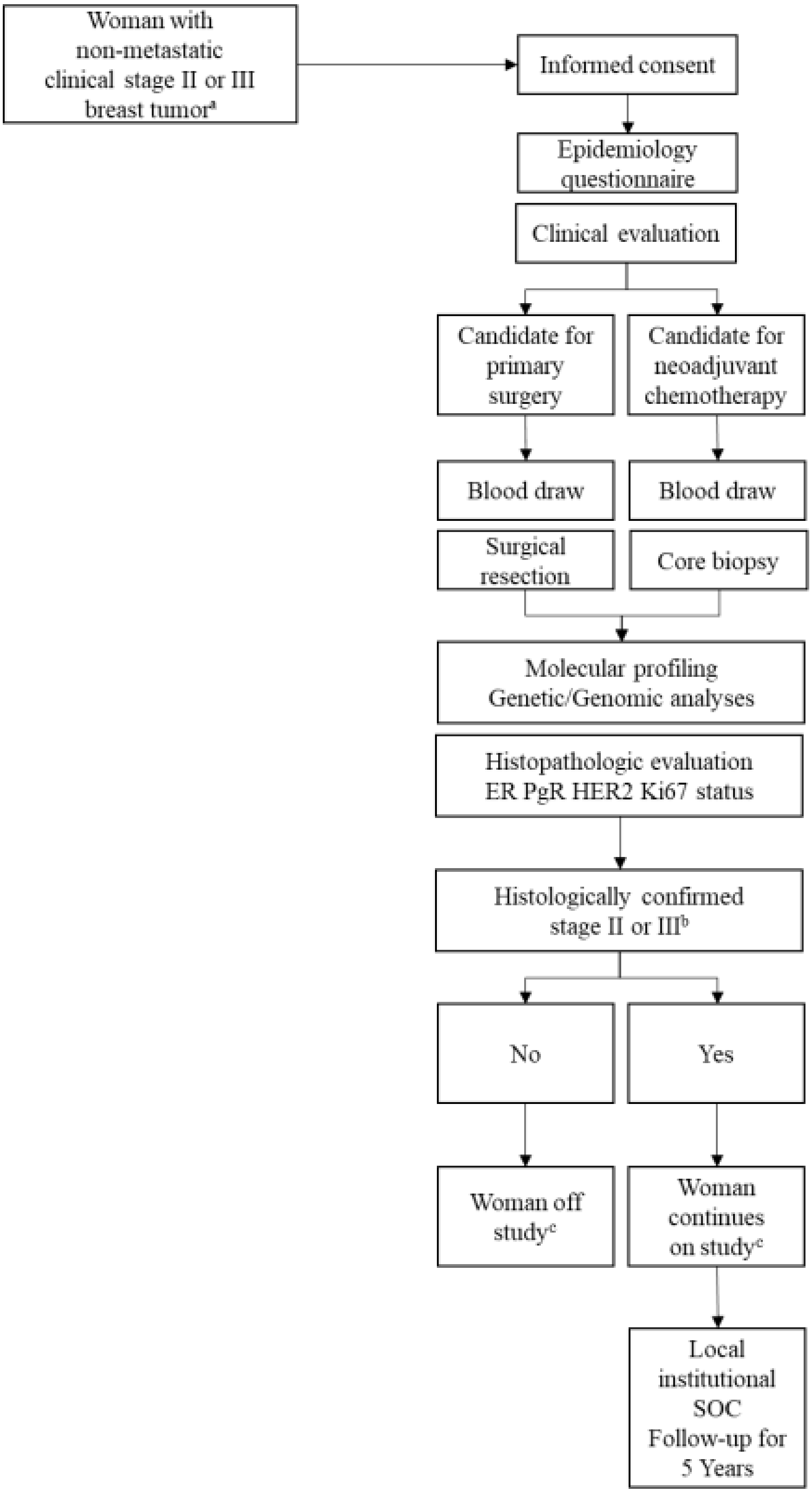

[a] Woman must also meet other inclusion/exclusion criteria
[b] Woman must also meet other standard of care treatment (SOC) inclusion/exclusion criteria
[c] Both women on- and off-study continue receiving local institutional SOC treatment

SUPPLEMENTAL MATERIAL 3: EPIDEMIOLOGICAL QUESTIONNAIRE OF LACRN BREAST CANCER STUDY

USLACRN MOLECULAR PROFILING OF BREAST CANCER STUDY

SURVEY IDENTIFICATION

Survey number: |__|__|__|__|__|

Center Name: ______________________________________ |__|__|__|
Interviewer: ______________________________________ |__|__|
Date interview started: |__|__| / |__|__| / |__|__|__|__|
Hour started: |__|__|:|__|__|
Data Entry: ______________________________________ |__|__|

SOCIOECONOMIC AND DEMOGRAPHIC MODULE

IDENTIFICATION OF INTERVIEWEE

1. Interviewee Identification Code
|__|__|__|__|/|__|__|__|__|/ |__|__|__|__|
(Country Code/Center Code/Individual Code)

2. How old are you?
|__|__| years

3. What country were you born in?
______________________________ |__|__|

4. How long have you been living in ....*?
***HERE EACH PARTICIPATING COUNTRY WILL ASK ABOUT THE COUNTRY WHERE THE STUDY IS BEING CONDUCTED.**
|__|__| years |__|__| months ***(Does not know: years = 99; months = 99)***

5. What ....* were you born in?
***HERE EACH COUNTRY WILL ASK IN TERMS OF THE COUNTRY'S POLITICO-ADMINISTRATIVE GEOGRAPHIC AREAS. FOR EXAMPLE, "IN WHAT PROVINCE" WILL BE ASKED IN ARGENTINA AND "IN WHAT STATE" WILL BE ASKED IN BRAZIL.**
______________________________ |__|__|

6. What city were you born in?
______________________________ |__|__|

7. Which of the following alternatives best describes your "geographic ancestry"? (Indicate the number(s) corresponding to the appropriate category)

   1. |__| African
   2. |__| European
   3. |__| Indigenous or Native American *(e.g., Native North and Central America, Native South American, Canadian First Peoples)*
   4. |__| Southwest Asian *(e.g., Turkey, Arabia, Iran, Syria)*
   5. |__| South Asian *(e.g., Pakistan and India)*
   6. |__| East and South East Asian *(e.g., China, Japan, Korea, Vietnam, Malaysia)*
   7. |__| Pacific Islander *(e.g., Native Hawaiian, Samoan, Maori, Aboriginal Australian)*
   8. |__| Not reported/unknown

8. What is your marital status?
1. |__| Married
2. |__| Consensual union (living together for more than one year)
3. |__| Separated / divorced
4. |__| Single
5. |__| Widowed

9. What is your current occupation? |__|__|__|__|
Code using the table form CIOU-88, International Classification of Occupations

10. What was your occupation for most of your life? |__|__|__|__|
Code using the table form CIOU-88, International Classification of Occupations

11. How many rooms does your house have? Including bathrooms and kitchen (that is, enclosed in walls and with a roof)
Interviewer: Does not know = 99
|__|__| rooms

12. How many of these rooms in your house are permanently used for sleeping?
Interviewer: Does not know = 99
|__|__| rooms

13. How many persons usually live in your house?
Interviewer: Does not ***know = 99***
|__|__| persons

14. In relation to public health services, which of the following do you have access to?
    a) WATER
       1) |__|__| Flowing/Potable Water
       2) |__|__| Pump
       3) |__|__| Motor
       4) |__|__| River
       5) |__|__| Laguna

    b) SANITATION
       1) |__|__| Sewer
       2) |__|__| Well
       3) |__|__| Septic tank

15. What is the total income of your family per month?
$ |__||__|.|__|__|__|,|__|__|

16. A total of how many persons are financially dependent on this monthly income?
Interviewer: Does not know = 99
|__|__| persons

17. Do you know how to:
    a) Read?
       1) |__| Yes
       2) |__| No

    b) Write?
       1) |__| Yes
       2) |__| No

18. Are you currently going to school?
    1. |__| Yes
    2. |__| No

In total, how many years of schooling have you completed?

19. ... in elementary school or *EGB*?	|__|__| years ***(does not know = 99)***
20. ... in secondary school?	|__|__| years ***(does not know = 99)***
21. ... in third level non university	|__|__| years ***(does not know = 99)***
22. ... in the university	|__|__| years ***(does not know = 99)***

23. Evaluation of module:
    1. |__| Unsatisfactory
    2. |__| Questionable
    3. |__| Reliable

MODULE FOR ACCESS TO HEALTH SERVICES

QUESTIONS MARKED WITH (**) ARE ONLY FOR THOSE PATIENTS WHO HAD A BIOPSY DONE AT ANOTHER INSTITUTION BEFORE COMING TO THE INSTITUTION WHERE THIS PROTOCOL IS BEING CONDUCTED OR STAGE IIB OR III PARTICIPANTS WHO HAD A BIOSPY DONE BEFORE SURGERY.

1. Do you have access to, or are you eligible for, any social health insurance (obra social), private health insurance, or work-related health insurance?
   1. |__| Yes
   2. |__| No ***(go to 5)***

2. Does this health insurance give you the right to office medical visits?
   1. |__| Yes
   2. |__| No
   9. |__| Does not know

3. Does this health insurance give you the right to additional examinations or studies?
   1. |__| Yes
   2. |__| No
   9. |__| Does not know

4. Does this health insurance give you the right to cancer treatments?
   1. |__| Yes
   2. |__| No
   9. |__| Does not know

The Papanicolau or PAP test is an examination to prevent specific diseases of the cervix; for this examination, material from the cervix is obtained for laboratory tests. This material is used to diagnose problems that may lead to cancer.

5. Have you ever had this type of uterine examination, that is, have you ever had a PAP test?
   1. |__| Yes
   2. |__| No ***(go to 9)***
   3. |__| Does not know ***(go to 9)***

6. When was the first time you had it?
Interviewer: does not know = 9999 and go to 7
   Year: |__|__|__|__|

7. How old were you when you had it done for the first time?
Interviewer: does not know = 99
   |__|__| Years

8. In general, how often do you have a PAP test done?
   1. |__| 1 or more times per year
   2. |__| 1 time every 2 years
   3. |__| 1 time every 3 years
   4. |__| Not regularly
   9. |__| Does not know

A clinical breast examination is one in which the doctor or nurse palpates your breasts to determine any possible problem, such as cysts, nodules or other possible diseases.

9. When was the first time that a doctor or nurse did a clinical breast examination on you?
Interviewer: does not know = 9999 and go to 10, otherwise go to *Erro! Fonte de referência não encontrada.*
   Year: |__|__|__|__|

10. How old were you the first time a doctor or nurse did a clinical breast examination on you?
Interviewer: does not know = 99
   |__|__| years

11. In general, how often do you have a breast examination done by a doctor or nurse?
1. |__| 1 or more times per year
2. |__| 1 time every 2 years
3. |__| 1 time every 3 years
4. |__| Not regularly
9. |__| Does not know

A mammography is an examination that women have done at a clinic. It is a specific radiological examination of the breasts. This examination is used to detect cysts, nodules, cancer or other breast diseases.

Interviewer: All clinical data must be extracted from Patient Medical History. Only use the module below if the clinical data cannot be obtained from the patient medical history or record.

12. When was the first time in your life that you had a mammography done?
Interviewer: Does not know = 9999
Year: |__|__|__|__|

13. How old were you when you had your first mammography done?
Interviewer: does not know = 99
|__|__| years

14. How often do you usually have a mammography?
1. |__| every 6 months
2. |__| once per year
3. |__| once every two years
4. |__| no regular intervals
9. |__| does not know

15. When did you have your last mammography?
Interviewer: Does not know = 9999
Year: |__|__|__|__|

16. How old were you when you had your last mammography?
Interviewer: Does not know = 99
|__|__| year

17. How many of your mammographies were reported as abnormal?
Interviewer: Does not know = 99
|__|__| mammographies

18. How many mammographies have you had in total?
Interviewer: Does not know = 99
|__|__| mammographies

19. Have you had any breast surgery, for any reason, inlcuding removal of any nodule or cyst or breast augmentation/reduction?
1. |__| Yes
2. |__| No ***(go to 23)***
9. |__| Does not know ***(go to 23)***

20. How many breast surgeries have you had?
|__|__| Surgeries

	21. How old were you when you had the ....?	22. What was done in this surgery? ***Interviewer: Can mark more than one choice.***	
1ª surgery	|___|___| years<br>***(does not know = 99)***<br>**o**<br>Año: |___|___|___|___| ***(does not know = 9999)***	1. Remove cysts/nodules or other benign lesions.<br>2 Breast augmentation.<br>3 Breast reduction.<br>4 Other (Specify____________________________________)<br>9 Does not know	☐<br>☐<br>☐<br>☐<br>☐
2ª surgery	|___|___| years<br>***(does not know = 99)***<br>**o**<br>Year: |___|___|___|___| ***(does not know = 9999)***	1. Remove cysts/nodules or other benign lesions.<br>2 Breast augmentation.<br>3 Breast reduction.<br>4 Other (Specify____________________________________)<br>9 Does not know	☐<br>☐<br>☐<br>☐<br>☐
3ª surgery	|___|___| years<br>***(does not know = 99)***<br>**o**<br>Year: |___|___|___|___| ***(does not know = 9999)***	1. Remove cysts/nodules or other benign lesions.<br>2 Breast augmentation.<br>3 Breast reduction.<br>4 Other (Specify____________________________________)<br>9 Does not know	☐<br>☐<br>☐<br>☐<br>☐

A breast biopsy is the removal of a sample of breast tissue to determine if the suspicious lesion is malignant (cancer) or benign (non-cancerous lesion)

23. Before the biopsy which has confirmed this diagnosis, have you already had this procedure done?
1. |__| Yes
2. |__| No ***(go to 26)***

24. How many breast biopsies did you have before presenting the breast disease that now brings you to the consultation?
|__|__| Biopsies

25. What was discovered in these biopsies?
Interviewer: Mark all the informed choices. If not known, mark the option does not know and leave the rest blank.

a,) 1ª biopsy	1. |__| Cyst<br>2. |__| Benign tumor<br>3. |__| Normal tissue (no problem)<br>4. |__| Malignant tumor<br>5. |__| Other (Specify:____________________________)
b.) 2ª biopsy	1. |__| Cyst<br>2. |__| Benign tumor<br>3. |__| Normal tumor (no problem)<br>4. |__| Malignant tumor<br>5. |__| Other (Specify:________________________)
c.) 3ª biopsy	1. |__| Cyst<br>2. |__| Benign tumor<br>3. |__| Normal tumor (no problem)<br>4. |__| Malignant tumor<br>5. |__| Other (Specify:________________________)
d.) 4ª biopsy	1. |__| Cyst<br>2. |__| Benign tumor<br>3. |__| Normal tumor (no problem)<br>4. |__| Malignant tumor<br>5. |__| Other (Specify:________________________)

Now we will discuss your current disease for which you came to this center for treatment.

26. How did you first discover this current problem in your breast?
1. |__| You yourself found this change in your breast. (go to 27)
2. |__| Your partner or other person discovered the change in your breast (go to 29)
3. |__| A doctor or other health professional discovered a change in your breast during a clinical examination (go to 29)
4. |__| It appeared in a routine mammography (go to 29)
9. |__| Does not remember

27. What change(s) did you discover in your breast?	28. When was the first time you felt/discovered this change? ***Interviewer: Does not know: 99 / 99 / 9999 go to 74. Otherwise go to 36***
1. |__| Nodule/cyst	Month: |__|__| Year: |__|__|__|__|
2. |__| Bloody or watery nipple secretion	Month: |__|__| Year: |__|__|__|__|
3. |__| Breast pain	Month: |__|__| Year: |__|__|__|__|
4. |__| Other(s):	
4.1 What ______________________	Month: |__|__| Year: |__|__|__|__|
4.2 What ______________________	Month: |__|__| Year: |__|__|__|__|
4.3 What______________________	Month: |__|__| Year: |__|__|__|__|

29. When was the first time you went to see a doctor about this change?
Interviewer: Does not know: Month = 99 and Year = 9999 and go to 38. Otherwise to go 35
Month: |__|__| Year: |__|__|__|__| **<YEAR OF FIRST CONSULTATION>**

30. Where was this consultation?
HERE EACH COUNTRY WILL BE ABLE TO ADAPT THE QUESTIONS THAT CORRESPOND TO ITS HEALTH SYSTEM
1. |__| Health post or center
2. |__| Private/prepaid medical office
3. |__| Emergency center
4. |__| Public hospital
5. |__| Home visit
6. |__| Other (Specify): ____

31. This health center where you received care was:
HERE EACH COUNTRY WILL BE ABLE TO ADAPT THE QUESTIONS THAT CORRESPOND TO ITS HEALTH SYSTEM
1. |__| Public
2. |__| Private/Health plan
3. |__| Charity/Foundation
9. |__| Does not know

32. How much time does it take you to go from your house to the place where you had the consultation?
|__|__| hours |__|__| minutes

33. Was the cost of this consultation covered, even in part, by any social health insurance (obra social), private health insurance, or work-related health insurance?
1. |__| Yes
2. |__| No
9. |__| Does not know

34. Did you pay any money directly for this consultation?
1. |__| Yes
2. |__| No
9. |__| Does not know

35. In this first consultation, did the doctor tell you to have a mammography done?
1. |__| Yes
2. |__| No ***(go to 44)***
9. |__| Does not know ***(go to 44)***

36. Were you able to have a mammography done the first time you contacted the health service to have it done?
1. |__| Yes
2 |__| No
9. |__| Does not know

37. How long after this first consultation did you have the mammography done?
Interviewer: Does not know: Months = 99 and Days = 99 and go to 52
|__|__| Months|__|__| Days <CALCULATE YEAR OF FIRST DIAG. MAMMOGRAPHY>

38. When was this mammography done?
Interviewer: Does not know: Month = 99 and Year = 9999 and go to 51. Otherwise go to 52
Month: |__|__| Year: |__|__|__|__| <YEAR OF FIRST DIAG. MAMMOGRAPHY>

39. In which health service did you have this first mammography?
HERE EACH COUNTRY WILL BE ABLE TO ADAPT THE QUESTIONS THAT CORRESPOND TO ITS HEALTH SYSTEM
1. |__| Health post or center
2. |__| Private clinic
3. |__| Laboratory
4. |__| Public hospital
5. |__| Mobile mammography imaging center

40. The place where you had this mammography was:
HERE EACH COUNTRY WILL BE ABLE TO ADAPT THE QUESTIONS THAT CORRESPOND TO ITS HEALTH SYSTEM
1. |__| Public
2. |__| Private/ Health plan
3. |__| Charity / Foundation
9. |__| Does not know

41. Was the cost of this mammography covered, even in part, by any social work or private study plan?
1. |__| Yes
2. |__| No
9. |__| Does not know

42. Did you pay for this mammogrpahy directly from your own pocket?
1. |__| Yes
2. |__| No
9. |__| Does not know

43. How much time does it take you to go from your house to the place where the mammography was done?
|__|__| hours |__|__| minutes

44. **When did you have the last mammography before the biopsy that confirmed your current diagnosis?
Interviewer: Does not know: Month = 99 and Year = 9999
Month: |__|__| Year: |__|__|__|__|

45. When did you receive instructions to come to this center for treatment for your current diagnosis?
Interviewer: Does not know: Month = 99 and Year = 9999
Month: |__|__| Year: |__|__|__|__| <YEAR OF LAST CONSULTATION>

46. ** When did you have the biopsy done that confirmed your current diagnosis?
Interviewer: Does not know: Month = 99 and Year = 9999
Month: |__|__| Year: |__|__|__|__|

47. **Were you able to have the biopsy done the first time you went to the health service to do it?
1. |__| Yes
2. |__| No
9. |__| Does not know

48. **Your biopsy was done at which of the following services?
HERE EACH COUNTRY WILL BE ABLE TO ADAPT THE QUESTIONS THAT CORRESPOND TO ITS HEALTH SYSTEM
1. |__| Health post or center
2. |__| Private cllinic
3. |__| Laboratory
4. |__| Public hospital
5. |__| Other (Specify): ____

49. **The place where your biopsy was done was :
HERE EACH COUNTRY WILL BE ABLE TO ADAPT THE QUESTIONS THAT CORRESPOND TO ITS HEALTH SYSTEM
1. |__| Public
2. |__| Private/ Health plan
3. |__| Charity / Foundation
9. |__| Does not know

50. **Was the cost of this biopsy covered, even in part, by any private health plan or social work?
1. |__| Yes
2. |__| No
9. |__| Does not know

51. **Did you pay any money directly from your pocket for this biopsy?
1. |__| Yes
2. |__| No
9. |__| Does not know

52. **How much time does it take you to go from your house to the place where the biopsy was done?
Interviewer: indicate in units of minutes or hours
|__|__| hours |__|__| minutes

53. **When did you receive the result of this biopsy?
Interviewer: Does not know: Month = 99 and Year = 9999 and go to the next module.
Mes: |__|__| Año: |__|__|__|__|

54. **How long after the biopsy was done did you receive the result?
Interviewer: Does not know: Months = 99 and Days = 99 and go to the following module.
|__|__| Months |__|__| days

55. Evaluation of ACCESS TO HEALTH Module
1. |__| Unsatisfactory
2. |__| Questionable
3. |__| Reliable

CANCER HISTORY MODULE OF INTERVIEWEE AND FAMILY

The following questions are about the interviewee's past and present relatives and family members and if they have or have had cancer. Please include only relatives related by blood. Do not include adoptive parents, grandparents, siblings or children.

1. Were you born from a multiple birth?
1. |__| Yes
2. |__| No ***(go to 5)***
9. |__| Does not know ***(go to 5)***

2. In total, how many babies were born from this pregnancy? Also take into account those stillborn.
Interviewer: Does not know = 99
|__|__| Babies

3. Of these siblings, how many were identical to you?
Interviewer: Does not know = 99. Do not forget to take into account those stillborn.
|___|___| Twins

4. Of these siblings, how many were not identical to you (non-identical twins)?
Interviewer: Does not know = 99
|___|___| Non-identical twins

5. Are you adopted?
1. |__| Yes
2. |__| No
9. |__| Does not know

6. Do you know the family history of your biological parents?
1. |__| Yes, both mother and father
2. |__| Yes, only mother
3. |__| Yes, only father
9. |__| Does not know ***(go to 56)***

7. Are your parents consanguinous, that is, are they related by blood ?
1. |__| Yes
2. |__| No
9. |__| Does not know

THE FOLLOWING QUESTIONS ARE ABOUT YOUR MOTHER

8. Is your mother still alive?
   1. |__| Yes ***(go to 10)***
   2. |__| No
   9. |__| Does not know ***(go to 10)***

9. How old is she now or how old was she when she died?
Interviewer: Does not know = 999
   |__|__|__| Years

10. What country was she born in?
   ______________________________________ |___|___|___| Country code

11. Which of the following alternatives best describes your mother's "geographic ancestry"? (Indicate the number(s) corresponding to the appropriate category)

   1. |__| African
   2. |__| European
   3. |__| Indigenous or Native American *(e.g., Native North and Central America, Native South American, Canadian First Peoples)*
   4. |__| Southwest Asian *(e.g., Turkey, Arabia, Iran, Syria)*
   5. |__| South Asian *(e.g., Pakistan and India)*
   6. |__| East and South East Asian *(e.g., China, Japan, Korea, Vietnam, Malaysia)*
   7. |__| Pacific Islander *(e.g., Native Hawaiian, Samoan, Maori, Aboriginal Australian)*
   8. |__| Not reported/unknown

12. Does/did she have any malignant tumor or cancer diagnosed by a doctor?
   1. |__| Yes
   2. |__| No ***(go to 16)***
   9. |__| Does not know ***(go to 16)***

13. How many diagnoses of cancer or malignant tumor has she had?
Interviewer: Does not know = 99
   |__|__| Diagnoses

	14. How old was she when the tumor was diagnosed? ***Int: If age = 99, ask the year it was diagnosed.***	15. What type of tumor did she have?
1st cancer	|__|__| Years<br>***Does not know = 99 <u>or</u>***<br>|__|__|__|__| Year diagnosed<br>***Does not know = 9999***	|__|__|
2nd cancer	|__|__| Years<br>***Does not know = 99 <u>or</u>***<br>|__|__|__|__| Year diagnosed<br>***Does not know = 9999***	|__|__|
3rd cancer	|__|__| Years<br>***Does not know = 99 <u>or</u>***<br>|__|__|__|__| Year diagnosed<br>***Does not know = 9999***	|__|__|

Tumor Code:

**Abdomen: AB**	**Adrenals: AD**	**Breast: BR**	**Chest wall: CW**	**CNS: CN**
**Heart: HT**	**Liver: LV**	**Lymph nodes: LN**	**Pancreas: PA**	**Peritoneum: PT**
**Skin: SK**	**Stomach:ST**	**Kidney: KD**	**Bone:BN**	**Other: OT**

THE FOLLOWING QUESTIONS ARE ABOUT YOUR FATHER

16. Is your father still alive?
   1. |__| Yes ***(go to 18)***
   2. |__| No
   9. |__| Does not know ***(go to 18)***

17. How old was he when he died?
Interviewer: Does not know = 999
   |__|__|__| Years

18. What country was he born in?

____ |__|__|__| Country code

19. Which of the following alternatives best describes your father's "geographic ancestry"? (Indicate the number(s) corresponding to the appropriate category)

1. |__| African
2. |__| European
3. |__| Indigenous or Native American *(e.g., Native North and Central America, Native South American, Canadian First Peoples)*
4. |__| Southwest Asian *(e.g., Turkey, Arabia, Iran, Syria)*
5. |__| South Asian *(e.g., Pakistan and India)*
6. |__| East and South East Asian *(e.g., China, Japan, Korea, Vietnam, Malaysia)*
7. |__| Pacific Islander *(e.g., Native Hawaiian, Samoan, Maori, Aboriginal Australian)*
8. |__| Not reported/unknown

20. Does/did he have any malignant tumor or cancer diagnosed by a doctor?
1. |__| Yes
2. |__| No ***(go to 24)***
9. |__| Does not know ***(go to 24)***

21. How many diagnoses of cancer or malignant tumor did he have?
Interviewer: Does not know = 99
|__|__| Diagnoses

	22. How old was he when the tumor was diagnosed? ***Int: If age = 99, ask the year he was diagnosed.***	23. What type of tumor does/did he have?
1st cancer	|__|__| Years<br>***Does not know = 99 <u>or</u>***<br>|__|__|__|__| Year diagnosed<br>***Does not know = 9999***	|__|__|
2nd cancer	|__|__| Years<br>***Does not know = 99 <u>or</u>***<br>|__|__|__|__| Year diagnosed<br>***Does not know = 9999***	|__|__|
3rd cancer	|__|__| Years<br>***Does not know = 99 <u>or</u>***<br>|__|__|__|__| Year diagnosed<br>***Does not know = 9999***	|__|__|

Tumor Code:

**Abdomen: AB**	**Adrenals: AD**	**Breast: BR**	**Chest wall: CW**	**CNS: CN**
**Heart: HT**	**Liver: LV**	**Lymph nodes: LN**	**Pancreas: PA**	**Peritoneum: PT**
**Skin: SK**	**Stomach:ST**	**Kidney: KD**	**Bone:BN**	**Other: OT**

Now I would like to know about YOUR grandparents. I will start with your maternal grandparents (your mother's mother and father) and then I will ask you about your paternal grandparents (your father's mother and father).

THE FOLLOWING QUESTIONS ARE ABOUT YOUR MATERNAL GRANDMOTHER

24. Is your maternal grandmother alive?
1. |__| Yes ***(go to 26)***
2. |__| No
9. |__| Does not know ***(go to 26)***

25. How old was she when she died?
Interviewer: Does not know = 999
|__|__|__| Years

26. What country was she born in?

____ |__|__|__| Country code

27. Which of the following alternatives best describes your maternal grandmother's "geographic ancestry"? (Indicate the number(s) corresponding to the appropriate category)

1. |__| African
2. |__| European
3. |__| Indigenous or Native American *(e.g., Native North and Central America, Native South American, Canadian First Peoples)*
4. |__| Southwest Asian *(e.g., Turkey, Arabia, Iran, Syria)*
5. |__| South Asian *(e.g., Pakistan and India)*
6. |__| East and South East Asian *(e.g., China, Japan, Korea, Vietnam, Malaysia)*
7. |__| Pacific Islander *(e.g., Native Hawaiian, Samoan, Maori, Aboriginal Australian)*
8. |__| Not reported/unknown

28. Does/has she had any cancer or malignant tumor diagnosed by a doctor?
1. |__| Yes
2. |__| No ***(go to 32)***
9. |__| Does not know ***(go to 32)***

29. How many diagnoses of cancer or malignant tumor did she have?
Interviewer: Does not know = 99
|__|__| Diagnoses

	30. How old was she when the tumor was diagnosed? ***Int: If age = 99, ask the year it was diagnosed.***	31. What type of tumor did she have.
1st cancer	|__|__| Years<br>***Does not know = 99 <u>or</u>***<br>|__|__|__|__| Year diagnosed<br>***Does not know = 9999***	|__|__|
2nd cancer	|__|__| Years<br>***Does not know = 99 <u>or</u>***<br>|__|__|__|__| Year diagnosed<br>***Does not know = 9999***	|__|__|
3rd cancer	|__|__| Years<br>***Does not know = 99 <u>or</u>***<br>|__|__|__|__| Year diagnosed<br>***Does not know = 9999***	|__|__|

Tumor Code:

**Abdomen: AB**	**Adrenals: AD**	**Breast: BR**	**Chest wall: CW**	**CNS: CN**
**Heart: HT**	**Liver: LV**	**Lymph nodes: LN**	**Pancreas: PA**	**Peritoneum: PT**
**Skin: SK**	**Stomach:ST**	**Kidney: KD**	**Bone:BN**	**Other: OT**

THE FOLLOWING QUESTIONS ARE ABOUT YOUR MATERNAL GRANDFATHER

32. Is your maternal grandfather alive?
1. |__| Yes ***(go to 34)***
2. |__| No
9. |__| Does not know ***(go to 34)***

33. How old was he when he died?
Interviewer: Does not know = 999
|__|__|__| Years

34. What country was he born in?
____ |__|__|__| Country code

35. Which of the following alternatives best describes your maternal grandfather's "geographic ancestry"? (Indicate the number(s) corresponding to the appropriate category)

1. |__| African
2. |__| European
3. |__| Indigenous or Native American *(e.g., Native North and Central America, Native South American, Canadian First Peoples)*
4. |__| Southwest Asian *(e.g., Turkey, Arabia, Iran, Syria)*
5. |__| South Asian *(e.g., Pakistan and India)*
6. |__| East and South East Asian *(e.g., China, Japan, Korea, Vietnam, Malaysia)*
7. |__| Pacific Islander *(e.g., Native Hawaiian, Samoan, Maori, Aboriginal Australian)*
8. |__| Not reported/unknown

36. Did he have any cancer or malignant tumor diagnosed by a doctor?

1. |__| Yes
2. |__| No ***(go to 40)***
9. |__| Does not know ***(go to 40)***

37. How many diagnoses of cancer or malignant tumor did he have?

Interviewer: Does not know = 99

|__|__| Diagnoses

	38. How old was he when the tumor was diagnosed? ***Int: If age = 99, ask the year it was diagnosed.***	39. What type of tumor does/did he have
1st cancer	|__|__| Years<br>***Does not know = 99 <u>or</u>***<br>|__|__|__|__| Year diagnosed<br>***Does not know = 9999***	|__|__|
2nd cancer	|__|__| Years<br>***Does not know = 99 <u>or</u>***<br>|__|__|__|__| Year diagnosed<br>***Does not know = 9999***	|__|__|
3rd cancer	|__|__| Years<br>***Does not know = 99 <u>or</u>***<br>|__|__|__|__| Year diagnosed<br>***Does not know = 9999***	|__|__|

Tumor Code:

**Abdomen: AB**	**Adrenals: AD**	**Breast: BR**	**Chest wall: CW**	**CNS: CN**
**Heart: HT**	**Liver: LV**	**Lymph nodes: LN**	**Pancreas: PA**	**Peritoneum: PT**
**Skin: SK**	**Stomach:ST**	**Kidney: KD**	**Bone:BN**	**Other: OT**

THE FOLLOWING QUESTIONS ARE ABOUT YOUR PATERNAL GRANDMOTHER

40. Is your paternal grandmother alive?

1. |__| Yes ***(go to 42)***
2. |__| No
9. |__| Does not know ***(go to 42)***

41. How old was she when she died?

Interviewer: Does not know = 999

|__|__|__| Years

42. What country was she born in?

______________________________ |__|__|__| Country code

43. Which of the following alternatives best describes your paternal grandmother's "geographic ancestry"? (Indicate the number(s) corresponding to the appropriate category)

1. |__| African
2. |__| European
3. |__| Indigenous or Native American *(e.g., Native North and Central America, Native South American, Canadian First Peoples)*
4. |__| Southwest Asian *(e.g., Turkey, Arabia, Iran, Syria)*
5. |__| South Asian *(e.g., Pakistan and India)*
6. |__| East and South East Asian *(e.g., China, Japan, Korea, Vietnam, Malaysia)*
7. |__| Pacific Islander *(e.g., Native Hawaiian, Samoan, Maori, Aboriginal Australian)*
8. |__| Not reported/unknown

44. Does/did she have any cancer or malignant tumor diagnosed by a doctor?

1. |__| Yes
2. |__| No ***(go to 48)***
9. |__| Does not know ***(go to 48)***

45. How may diagnoses of cancer or malignant tumor did she have?

Interviewer: Does not know = 99

|__|__| Diagnoses

	46. How old was she when the tumor was diagnosed? ***Int: If age = 99, ask the year it was diagnosed.***	47. What type of tumor did she have
1st cancer	|__|__| Years<br>***Does not know = 99*** ***<u>or</u>***<br>|__|__|__|__| Year diagnosed<br>***Does not know = 9999***	|__|__|
2nd cancer	|__|__| Years<br>***Does not know = 99*** ***<u>or</u>***<br>|__|__|__|__| Year diagnosed<br>***Does not know = 9999***	|__|__|
3rd cancer	|__|__| Years<br>***Does not know = 99*** ***<u>or</u>***<br>|__|__|__|__| Year diagnosed<br>***Does not know = 9999***	|__|__|

Tumor Code:

**Abdomen: AB**	**Adrenals: AD**	**Breast: BR**	**Chest wall: CW**	**CNS: CN**
**Heart: HT**	**Liver: LV**	**Lymph nodes: LN**	**Pancreas: PA**	**Peritoneum: PT**
**Skin: SK**	**Stomach:ST**	**Kidney: KD**	**Bone:BN**	**Other: OT**

THE FOLLOWING QUESTIONS ARE ABOUT YOUR PATERNAL GRANDFATHER

48. Is your paternal grandfather alive?

1. |__| Yes ***(go to 50)***
2. |__| No
9. |__| Does not know ***(go to 50)***

49. How old was he when he died?

Interviewer: Does not know = 999

|__|__|__| Years

50. What country was he born in?

____ |__|__|__| Country code

51. Which of the following alternatives best describes your paternal grandfather's "geographic ancestry"? (Indicate the number(s) corresponding to the appropriate category)

1. |__| African
2. |__| European
3. |__| Indigenous or Native American *(e.g., Native North and Central America, Native South American, Canadian First Peoples)*
4. |__| Southwest Asian *(e.g., Turkey, Arabia, Iran, Syria)*
5. |__| South Asian *(e.g., Pakistan and India)*
6. |__| East and South East Asian *(e.g., China, Japan, Korea, Vietnam, Malaysia)*
7. |__| Pacific Islander *(e.g., Native Hawaiian, Samoan, Maori, Aboriginal Australian)*
8. |__| Not reported/unknown

52. Does/did he have any cancer or malignant tumor diagnosed by a doctor?

1. |__| Yes
2. |__| No ***(go to 56)***
9. |__| Does not know ***(go to 56)***

53. How may diagnoses of cancer or malignant tumor did he have?

Interviewer: Does not know = 99

|__|__| Diagnoses

	54. How old was he when the tumor was diagnosed? ***Int: If age = 99, ask the year it was diagnosed.***	55. What type of tumor does/did he have
1st cancer	|__|__| Years<br>***Does not know = 99*** **<u>or</u>**<br>|__|__|__|__| Year diagnosed<br><br>***Does not know = 9999***	|__|__|
2nd cancer	|__|__| Years<br>***Does not know = 99*** **<u>or</u>**<br>|__|__|__|__| Year diagnosed<br><br>***Does not know = 9999***	|__|__|
3rd cancer	|__|__| Years<br>***Does not know = 99*** **<u>or</u>**<br>|__|__|__|__| Year diagnosed<br><br>***Does not know = 9999***	|__|__|

Tumor Code:

**Abdomen: AB**	**Adrenals: AD**	**Breast: BR**	**Chest wall: CW**	**CNS: CN**
**Heart: HT**	**Liver: LV**	**Lymph nodes: LN**	**Pancreas: PA**	**Peritoneum: PT**
**Skin: SK**	**Stomach:ST**	**Kidney: KD**	**Bone:BN**	**Other: OT**

Now I would like to know about YOUR brothers and sisters. I will start with your brothers and sisters who are related to you by both your mother and father, and then I will ask you about your half brothers and sisters (related to you by either your mother or father).

THE FOLLOWING QUESTIONS ARE ABOUT YOUR BROTHERS AND SISTERS

56. How many brothers and sisters do/did you have? Please do not include half-brothers or half-sisters.

Interviewer: If no brothers or sisters = 00 and go to 62.

Does not know = 99

|__|__| sisters
|__|__| brothers

Starting with your oldest brother/sister, please answer the following questions: :

57. Sex?	58. Living?	59. How old is he/she now or how old was he/she when he/she died?	60. Did he/she have cancer?	61. What type of cancer does/did he/she have? ***Interviewer: In case he/she has/had more than one type of cancer, please list all.***	81. At what age was the cancer diagnosed?
1. |__| Female<br>2. |__| Male	1. |__| Living<br>2. |__| Deceased<br>9. |__| Does not know	|__|__| Years<br>***Does not know = 99***	1. |__| Yes<br>2. |__| No ***(Next)***<br>9. |__| Does not know ***(Next)***	|__|__|Cod. 1<br>|__|__|Cod. 2<br>|__|__|Cod. 3	|__|__| Years Ca1<br>|__|__| Years Ca2<br>|__|__| Years Ca3
1. |__| Female<br>2. |__| Male	1. |__| Living<br>2. |__| Deceased<br>9. |__| Does not know	|__|__| Years<br>***Does not know = 99***	1. |__| Yes<br>2. |__| No ***(Next)***<br>9. |__| Does not know ***(Next)***	|__|__|Cod. 1<br>|__|__|Cod. 2<br>|__|__|Cod. 3	|__|__| Years Ca1<br>|__|__| Years Ca2<br>|__|__| Years Ca3
1. |__| Female<br>2. |__| Male	1. |__| Living<br>2. |__| Deceased<br>9. |__| Does not know	|__|__| Years<br>***Does not know = 99***	1. |__| Yes<br>2. |__| No ***(Next)***<br>9. |__| Does not know ***(Next)***	|__|__|Cod. 1<br>|__|__|Cod. 2<br>|__|__|Cod. 3	|__|__| Years Ca1<br>|__|__| Years Ca2<br>|__|__| Years Ca3
1. |__| Female<br>2. |__| Male	1. |__| Living<br>2. |__| Deceased<br>9. |__| Does not know	|__|__| Years<br>***Does not know = 99***	1. |__| Yes<br>2. |__| No ***(Next)***<br>9. |__| Does not know ***(Next)***	|__|__|Cod. 1<br>|__|__|Cod. 2<br>|__|__|Cod. 3	|__|__| Years Ca1<br>|__|__| Years Ca2<br>|__|__| Years Ca3
1. |__| Female<br>2. |__| Male	1. |__| Living<br>2. |__| Deceased<br>9. |__| Does not know	|__|__| Years<br>***Does not know = 99***	1. |__| Yes<br>2. |__| No ***(Next)***<br>9. |__| Does not know ***(Next)***	|__|__|Cod. 1<br>|__|__|Cod. 2<br>|__|__|Cod. 3	|__|__| Years Ca1<br>|__|__| Years Ca2<br>|__|__| Years Ca3

Tumor Code:

**Abdomen: AB**	**Adrenals: AD**	**Breast: BR**	**Chest wall: CW**	**CNS: CN**
**Heart: HT**	**Liver: LV**	**Lymph nodes: LN**	**Pancreas: PA**	**Peritoneum: PT**
**Skin: SK**	**Stomach:ST**	**Kidney: KD**	**Bone:BN**	**Other: OT**

THE FOLLOWING QUESTIONS ARE ABOUT YOUR HALF-BROTHERS AND HALF-SISTERS

62. How many half-brothers and half-sisters do/did you have?
Interviewer: If no half-brothers or sisters = 00 and go to 69
Does not know = 99
|__|__| Half-sisters
|__|__| Half-brothers

Starting with your oldest half-brother/sister, please answer the following questions: :

63. What is your relation to this half-sibling?	64. Sex?	65. Living?	66. How old is he/she now or how old was he/she when he/she died?	67. Did he/she have cancer?	68. What type of cancer does/did he/she have? ***Interviewer: In case he/she has/had more than one type of cancer, please list all.***	81. At what age was the cancer diagnosed?
1. |__| Maternal<br>2. |__| Paternal	1. |__| Female<br>2. |__| Male	1. |__| Living<br>2. |__| Deceased<br>9. |__| Does not know	|__|__| Years<br>***Does not know = 99***	1. |__| Yes<br>2. |__| No ***(Next)***<br>9. |__| Does not know ***(Next)***	|__|__|Cod. 1<br>|__|__|Cod. 2<br>|__|__|Cod. 3	|__|__| Years Ca1<br>|__|__| Years Ca2<br>|__|__| Years Ca3
1. |__| Maternal<br>2. |__| Paternal	1. |__| Female<br>2. |__| Male	1. |__| Living<br>2. |__| Deceased<br>9. |__| Does not know	|__|__| Years<br>***Does not know = 99***	1. |__| Yes<br>2. |__| No ***(Next)***<br>9. |__| Does not know ***(Next)***	|__|__|Cod. 1<br>|__|__|Cod. 2<br>|__|__|Cod. 3	|__|__| Years Ca1<br>|__|__| Years Ca2<br>|__|__| Years Ca3
1. |__| Maternal<br>2. |__| Paternal	1. |__| Female<br>2. |__| Male	1. |__| Living<br>2. |__| Deceased<br>9. |__| Does not know	|__|__| Years<br>***Does not know = 99***	1. |__| Yes<br>2. |__| No ***(Next)***<br>9. |__| Does not know ***(Next)***	|__|__|Cod. 1<br>|__|__|Cod. 2<br>|__|__|Cod. 3	|__|__| Years Ca1<br>|__|__| Years Ca2<br>|__|__| Years Ca3
1. |__| Maternal<br>2. |__| Paternal	1. |__| Female<br>2. |__| Male	1. |__| Living<br>2. |__| Deceased<br>9. |__| Does not know	|__|__| Years<br>***Does not know = 99***	1. |__| Yes<br>2. |__| No ***(Next)***<br>9. |__| Does not know ***(Next)***	|__|__|Cod. 1<br>|__|__|Cod. 2<br>|__|__|Cod. 3	|__|__| Years Ca1<br>|__|__|Years Ca2<br>|__|__| Years Ca3

Tumor Code:

**Abdomen: AB**	**Adrenals: AD**	**Breast: BR**	**Chest wall: CW**	**CNS: CN**
**Heart: HT**	**Liver: LV**	**Lymph nodes: LN**	**Pancreas: PA**	**Peritoneum: PT**
**Skin: SK**	**Stomach:ST**	**Kidney: KD**	**Bone:BN**	**Other: OT**

THE FOLLOWING QUESTIONS ARE ABOUT YOUR CHILDREN

69. How many children do/did you have?
Interviewer: If no children = 00 and go to 75.
Does not know = 99
|__|__| daughters
|__|__| sons

Starting with your oldest child, please answer the following questions:

70. Sex?	71. Living?	72. How old is he/she now or how old was he/she when he/she died?	73. Does/did he/she have cancer?	74. What type of cancer does/did he/she have? ***Interviewer: In case he/she has/had more than one type of cancer, please list all.***	81. At what age was the cancer diagnosed?
1. |__| Female<br>2. |__| Male	1. |__| Living<br>2. |__| Deceased<br>9. |__| Does not know	|__|__| Years<br>***Does not know = 99***	1. |__| Yes<br>2. |__| No ***(Next)***<br>9. |__| Does not know ***(Next)***	|__|__|Cod. 1<br>|__|__|Cod. 2<br>|__|__|Cod. 3	|__|__| Years Ca1<br>|__|__| Years Ca2<br>|__|__| Years Ca3
1. |__| Female<br>2. |__| Male	1. |__| Living<br>2. |__| Deceased<br>9. |__| Does not know	|__|__| Years<br>***Does not know = 99***	1. |__| Yes<br>2. |__| No ***(Next)***<br>9. |__| Does not know ***(Next)***	|__|__|Cod. 1<br>|__|__|Cod. 2<br>|__|__|Cod. 3	|__|__| Years Ca1<br>|__|__| Years Ca2<br>|__|__| Years Ca3
1. |__| Female<br>2. |__| Male	1. |__| Living<br>2. |__| Deceased<br>9. |__| Does not know	|__|__| Years<br>***Does not know = 99***	1. |__| Yes<br>2. |__| No ***(Next)***<br>9. |__| Does not know ***(Next)***	|__|__|Cod. 1<br>|__|__|Cod. 2<br>|__|__|Cod. 3	|__|__| Years Ca1<br>|__|__| Years Ca2<br>|__|__| Years Ca3
1. |__| Female<br>2. |__| Male	1. |__| Living<br>2. |__| Deceased<br>9. |__| Does not know	|__|__| Years<br>***Does not know = 99***	1. |__| Yes<br>2. |__| No ***(Next)***<br>9. |__| Does not know ***(Next)***	|__|__|Cod. 1<br>|__|__|Cod. 2<br>|__|__|Cod. 3	|__|__| Years Ca1<br>|__|__| Years Ca2<br>|__|__| Years Ca3

Tumor Code:

**Abdomen: AB**	**Adrenals: AD**	**Breast: BR**	**Chest wall: CW**	**CNS: CN**
**Heart: HT**	**Liver: LV**	**Lymph nodes: LN**	**Pancreas: PA**	**Peritoneum: PT**
**Skin: SK**	**Stomach:ST**	**Kidney: KD**	**Bone:BN**	**Other: OT**

Now I would like to know about YOUR aunts and uncles. I will start with your maternal aunts and uncles (your mother's sisters and brothers) and then I will ask you about your paternal aunts and uncles (your father's sisters and brothers).

THE FOLLOWING QUESTIONS ARE ABOUT YOUR MATERNAL AUNTS AND UNCLES

75. How may aunts and uncles on your mother's side do/did you have?
Interviewer:If no aunts and uncles on mother's side = 00 and go to 81.
Does not know = 99

|__|__| maternal aunts
|__|__| maternal uncles

Starting with your oldest maternal aunt/uncle please answer the following questions:

76. Sex?	77. Living?	78. How old is he/she now or how old was he/she when he/she died?	79. Does/did he/she have cancer?	80. What type of cancer does/did he/she have? ***Interviewer: In case he/she has/had more than one type of cancer, please list all.***	81. At what age was the cancer diagnosed?
1. |__| Female<br>2. |__| Male	1. |__| Living<br>2. |__| Deceased<br>9. |__| Does not know	|__|__| Years<br>***Does not know = 99***	1. |__| Yes<br>2. |__| No ***(Next)***<br>9. |__| Does not know ***(Next)***	|__|__|Cod. 1<br>|__|__|Cod. 2<br>|__|__|Cod. 3	|__|__| Years Ca1<br>|__|__| Years Ca2<br>|__|__| Years Ca3
1. |__| Female<br>2. |__| Male	1. |__| Living<br>2. |__| Deceased<br>9. |__| Does not know	|__|__| Years<br>***Does not know = 99***	1. |__| Yes<br>2. |__| No ***(Next)***<br>9. |__| Does not know ***(Next)***	|__|__|Cod. 1<br>|__|__|Cod. 2<br>|__|__|Cod. 3	|__|__| Years Ca1<br>|__|__| Years Ca2<br>|__|__| Years Ca3
1. |__| Female<br>2. |__| Male	1. |__| Living<br>2. |__| Deceased<br>9. |__| Does not know	|__|__| Years<br>***Does not know = 99***	1. |__| Yes<br>2. |__| No ***(Next)***<br>9. |__| Does not know ***(Next)***	|__|__|Cod. 1<br>|__|__|Cod. 2<br>|__|__|Cod. 3	|__|__| Years Ca1<br>|__|__| Years Ca2<br>|__|__| Years Ca3
1. |__| Female<br>2. |__| Male	1. |__| Living<br>2. |__| Deceased<br>9. |__| Does not know	|__|__| Years<br>***Does not know = 99***	1. |__| Yes<br>2. |__| No ***(Next)***<br>9. |__| Does not know ***(Next)***	|__|__|Cod. 1<br>|__|__|Cod. 2<br>|__|__|Cod. 3	|__|__| Years Ca1<br>|__|__| Years Ca2<br>|__|__| Years Ca3

Tumor Code:

**Abdomen: AB**	**Adrenals: AD**	**Breast: BR**	**Chest wall: CW**	**CNS: CN**
**Heart: HT**	**Liver: LV**	**Lymph nodes: LN**	**Pancreas: PA**	**Peritoneum: PT**
**Skin: SK**	**Stomach:ST**	**Kidney: KD**	**Bone:BN**	**Other: OT**

THE FOLLOWING QUESTIONS ARE ABOUT YOUR PATERNAL AUNTS AND UNCLES

81. How many aunts and uncles on your father's side do/did you have?
Interviewer: If no aunts or uncles on father's side = 00 and go to the next module
Does not know = 99
|__|__| paternal aunts
|__|__| paternal uncles

Starting with your oldest paternal aunt/uncle please answer the following questions:

82. Sex?	83. Living?	84. How old is he/she now or how old was he/she when he/she died?	85. Does/did he/she have cancer?	86. What type of cancer does/did he/she have? ***Interviewer: In case he/she has/had more than one type of cancer, please list all.***	81. At what age was the cancer diagnosed?
1. |__| Female<br>2. |__| Male	1. |__| Living<br>2. |__| Deceased<br>9. |__| Does not know	|__|__| Years<br>***Does not know = 99***	1. |__| Yes<br>2. |__| No ***(Next)***<br>9. |__| Does not know ***(Next)***	|__|__|Cod. 1<br>|__|__|Cod. 2<br>|__|__|Cod. 3	|__|__| Years Ca1<br>|__|__| Years Ca2<br>|__|__| Years Ca3
1. |__| Female<br>2. |__| Male	1. |__| Living<br>2. |__| Deceased<br>9. |__| Does not know	|__|__| Years<br>***Does not know = 99***	1. |__| Yes<br>2. |__| No ***(Next)***<br>9. |__| Does not know ***(Next)***	|__|__|Cod. 1<br>|__|__|Cod. 2<br>|__|__|Cod. 3	|__|__| Years Ca1<br>|__|__| Years Ca2<br>|__|__| Years Ca3
1. |__| Female<br>2. |__| Male	1. |__| Living<br>2. |__| Deceased<br>9. |__| Does not know	|__|__| Years<br>***Does not know = 99***	1. |__| Yes<br>2. |__| No ***(Next)***<br>9. |__| Does not know ***(Next)***	|__|__|Cod. 1<br>|__|__|Cod. 2<br>|__|__|Cod. 3	|__|__| Years Ca1<br>|__|__| Years Ca2<br>|__|__| Years Ca3
1. |__| Female<br>2. |__| Male	1. |__| Living<br>2. |__| Deceased<br>9. |__| Does not know	|__|__| Years<br>***Does not know = 99***	1. |__| Yes<br>2. |__| No ***(Next)***<br>9. |__| Does not know ***(Next)***	|__|__|Cod. 1<br>|__|__|Cod. 2<br>|__|__|Cod. 3	|__|__| Years Ca1<br>|__|__| Years Ca2<br>|__|__| Years Ca3

Tumor Code:

**Abdomen: AB**	**Adrenals: AD**	**Breast: BR**	**Chest wall: CW**	**CNS: CN**
**Heart: HT**	**Liver: LV**	**Lymph nodes: LN**	**Pancreas: PA**	**Peritoneum: PT**
**Skin: SK**	**Stomach:ST**	**Kidney: KD**	**Bone:BN**	**Other: OT**

HORMONAL AND REPRODUCTIVE HISTORY MODULE

The following questions are about your menstrual periods, pregnancies, menopause and hormones that you take or have taken.

1. How old were you when you had your first menstrual period?

Interviewer: Does not know = 99

|__|__| Years

2. Considering all your pregnancies – live births or stillbirths, miscarriages, tubal pregnancy, how many times have you been pregnant?

Interviewer: Does not know = 99

|__|__| times

Now I would like to learn details about each one of your pregnancies.

Interviewer: ask questions d4 to d9 for each pregnancy in chronological order, starting with the first pregnancy up to the most recent one. Ask all the questions about one pregnancy before going on to the next one.

The number of pregnancies in the Table will depend on the number of times you have been pregnant, that is, there is no predefined limit.

	1st PREGNANCY	2nd PREGNANCY	3rd PREGNANCY
3. How old were you when you became pregnant, including live births or stillbirths, tubal pregnancy, possible miscarriages?	|__|__| Years ***(does not know = 99)***	|__|__| Years ***(does not know = 99)***	|__|__| Years ***(does not know = 99)***
4. How many babies were born:	|__|__| Live<br>|__|__| Dead	|__|__| Live<br>|__|__| Dead	|__|__| Live<br>|__|__| Dead
5. How long did this pregnancy last?	|__|__| Weeks ***(does not know = 99) If in question 6, live=0 and dead > 1 or = 1, go to NEXT PREGNANCY or go to 10***	|__|__| Weeks ***(does not know = 99) If in question 6, live=0 and dead > 1 or = 1, go to NEXT PREGNANCY or go to 10***	|__|__| Weeks ***(does not know = 99) If in question 6, live=0 and dead > 1 or = 1, go to NEXT PREGNANCY or go to 10***
6. Did you breast-feed this baby (or these babies), even if for a short time?	1. |__| Yes<br>2. |__| No ***(go to next preg. or go to 10)***<br>9. |__| Does not know ***(go to next preg. or go to 10)***.	1. |__| Yes<br>2. |__| No ***(go to next preg. or go to 10)***<br>9. |__| Does not know ***(go to next preg. or go to 10)***.	1. |__| Yes<br>2. |__| No ***(go to next preg. or go to 10)***<br>9. |__| Does not know ***(go to next preg. or go to 10)***.
7. For how long did you breast-feed this baby (or these babies)?	|__|__| Years<br>|__|__| Months<br>***< 1 month = 00***<br>***Does not know = 99***<br>**go to next preg. or go to 10**	|__|__| Years<br>|__|__| Months<br>***< 1 month = 00***<br>***Does not know = 99***<br>**go to next preg. or go to 10**	|__|__| Years<br>|__|__| Months<br>***< 1 month = 00***<br>***Does not know = 99***<br>**go to next preg. or go to 10**

8. Have you ever undergone treatments to become pregnant or maintain a pregnancy?
   1. |__| Yes
   2. |__| No ***(go to 10)***
   9. |__| Does not know ***(go to 10)***

9. What treatment did you do for this?

Interviewer: Read each item and mark Yes, No, or Do not know.

	Yes	No	Does not know
1. *In vitro* fertilization?	|__|	|__|	|__|
2. Artificial insemination?	|__|	|__|	|__|
3. Hormone treatment? (Specify: ________________________)	|__|	|__|	|__|
4. Any other treatment? (Specify: ________________________)	|__|	|__|	|__|

The following questions are about some methods to avoid pregnancy and hormones that you would have used or are using.

Interviewer: the next questions are about the hormonal birth control methods that the patient could have used. We are not interested in the methods such as condoms, diaphragm, sperm gel, etc.

10. Did you take BIRTH CONTROL PILLS to avoid pregnancy or for any other reason, such as irregular menstrual cycle, acne, colics, endometriosis or polycystic ovary?
    1. |__| Yes, I used them
    2. |__| Yes, I am currently using them ***(go to 12)***
    3. |__| I never used them ***(go to 13)***

11. How long ago did you stop using birth control pills?

Interviewer: Read the choices.

1. |__| Less than 1 year
2. |__| From 1 to 4 years
3. |__| From 5 to 9 years
4. |__| More than 10 years
9. |__| Does not know

12. Adding up all the periods of time you used birth control pills, for any reason, how long did you use them in total? Exclude periods when you were not using them.
    Interviewer: Help the informant add up all the periods of time when she was using the birth control pills and excluding those when she did not use them.
    Interviewer: Does not know: Years = 99 and Months = 99; < 1 Month = 00
    |__|__| Years and |__|__| Months

Now I will read a list of other methods to avoid pregnancy that you may have used. Please answer if you have used them, are using them now, or never have used them. For each method you list, please indicate the length of time you used it.

13. Have you used, are you now using or did you never use ....?

Interviewer: ask each method, in case the answer is that she used it or is now using it to go d17. Otherwise, go to the next method or 18.

		14. For how long did you use/have you been using ...? Do not include periods of time when you did not use.
a. Hormone injection	1. |__| I used them ***(go to 15)***<br>2. |__| I am now using ***(GO TO 15)***<br>3. |__| I never used<br>9. |__| Does not know	|__|__| and |__|__| Years and Months<br>***< 1 month = 00 and 00***<br>***Does not know = 99 and 00***
b. Hormone implant (including adhesive/patches)	1. |__| I used them ***(go to 15)***<br>2. |__| I am now using ***(GO TO 15)***<br>3. |__| I never used<br>9. |__| Does not know	|__|__| and |__|__| Years and Months<br>***< 1 month = 00 and 00***<br>***Does not know = 99 and 00***
c.Vaginal ring (Nuvaring)	1. |__| I used them ***(go to 15)***<br>2. |__| I am now using ***(GO TO 15)***<br>3. |__| I never used<br>9. |__| Does not know	|__|__| and |__|__| Years and Months<br>***< 1 month = 00 and 00***<br>***Does not know = 99 and 00***
d. Hormonal IUD (Mirena)	1. |__| I used them ***(go to 15)***<br>2. |__| I am now using ***(GO TO 15)***<br>3. |__| I never used<br>9. |__| Does not know	|__|__| and |__|__| Years and Months<br>***< 1 month = 00 and 00***<br>***Does not know = 99 and 00***

15. Are you still menstruating?
    1. |__| Yes ***(go to 18)***
    3. |__| No
    9. |__| Does not know

16. How many years ago did you last menstruate?
    |__|__| Years ***(does not know = 99)***

17. Why did you no longer menstruate?

Interviewer: Read each item and mark ONLY one choice.

1. |__| Menstruation stopped naturally.
2. |__| Menstruation stopped due to surgery for removal of uterus or ovaries
3. |__| Menstruation stopped due to radiation therapy or chemotherapy
4. |__| Other (Specific: ____)
9. |__| Does not know.

To treat menopause symptoms, sometimes doctors prescribe drugs that are hormones. These drugs, termed replacement hormones, can be used as pills, injections, adhesive patches, gel to place on body or vaginal creams.

18. Have you at any time used hormone treatment for menopause symptoms (hormone replacement)?
    1. |__| Yes
    2. |__| No ***(go to 22)***
    9. |__| Does not know ***(go to 22)***

19. What did you use?

Interviewer: Read each item and mark Yes, No, or Does not know.

	Yes	No	Does not know
1. Pill	|__|	|__|	|__|
2. Patch or adhesive	|__|	|__|	|__|
3. Gel	|__|	|__|	|__|
4. Cream	|__|	|__|	|__|
5. Other? Specify: ______________________________	|__|	|__|	|__|

Interviewer: if 19.1 = yes, go to 20. Otherwise go to 22.

20. Do you know if the pill you took was only estrogen, only progesterone or a combination of the two or did you take two types of pills?
    1. |__| Estrogen pill
    2. |__| Progesterone pill
    3. |__| Combined estrogen and progesterone pill
    4. |__| Two pills: one estrogen and the other progesterone
    9. |__| Does not know.

21. Adding up all the time you used these drugs, what was the total time you used them? Exclude the periods of time that you normally did not use them.

Interviewer Help the informant to add up all the periods of time that she used replacement hormones and exclude those during which she did not use them.

Interviewer: Does not know: Years = 99 and Months = 99; < 1 Month = 00

|__|__| Years and |__|__| Months

22. Evaluation of hormonal and reproductive history:
    1. |__| Unsatisfactory
    2. |__| Questionable
    3. |__| Reliable

MEDICAL HISTORY MODULE

The following questions are about certain health diseases or disorders that you have or have had. (Interviewers: All clinical data must be extracted from patient medical history. Only use this module below if the clinical data cannot be obtained from the patient medical history or record.)

Has any doctor told you that you have or have had any of the following health disorders?

Interviewer: ask about each disorder. If she has one of them, go to 2. Otherwise, go to the next disorder

		1. What year were you diagnosed?	2. As a result of this disorder, were you hospitalized, did you have surgery or was some medicine suggested?
a. Arterial hypertension/High blood pressure	1. |__| Yes<br>2. |__| No<br>9. |__| Does not know	|__|___| Age<br>***Does not know = 99 or***<br>|__|__|__|__| Year diagnosed<br>***Does not know = 9999***	1. |__|__| Hospitalized<br>2. |__|__| Surgery<br>3. |__|__| Drug<br>4. |__|__| No treatment
b. Diabetes mellitus/blood sugar	1. |__| Yes<br>2. |__| No<br>9. |__| Does not know	|__|___| Age<br>***Does not know = 99 or***<br>|__|__|__|__| Year diagnosed<br>***Does not know = 9999***	1. |__|__| Hospitalized<br>2. |__|__| Surgery<br>3. |__|__| Medication<br>4. |__|__| No treatment
c. Hypercholesterolemia/ High cholesterol	1. |__| Yes<br>2. |__| No<br>9. |__| Does not know	|__|___| Age<br>***Does not know = 99 or***<br>|__|__|__|__| Year diagnosed<br>***Does not know = 9999***	1. |__|__| Hospitalized<br>2. |__|__| Surgery<br>3. |__|__| Medication<br>4. |__|__| No treatment
d. Asthma	1. |__| Yes<br>2. |__| No<br>9. |__| Does not know	|__|___| Age<br>***Does not know = 99 or***<br>|__|__|__|__| Year diagnosed<br>***Does not know = 9999***	1. |__|__| Hospitalized<br>2. |__|__| Surgery<br>3. |__|__| Medication<br>4. |__|__| No treatment

		1. What year were you diagnosed?	2. As a result of this disorder, were you hospitalized, did you have surgery or was some medicine suggested?
f. EPOC/emphysema	1. |__| Yes<br>2. |__| No<br>9. |__| Does not know	|__|___| Age<br>***Does not know = 99 <u>or</u>***<br>|__|__|__|__| Year diagnosed<br>***Does not know = 9999***	1. |__|__| Hospitalized<br>2. |__|__| Surgery<br>3. |__|__| Medication<br>4. |__|__| No treatment
g. Epilepsy	1. |__| Yes<br>2. |__| No<br>9. |__| Does not know	|__|___| Age<br>***Does not know = 99 <u>or</u>***<br>|__|__|__|__| Year diagnosed<br>***Does not know = 9999***	1. |__|__| Hospitalized<br>2. |__|__| Surgery<br>3. |__|__| Medication<br>4. |__|__| No treatment
i. Cardiac failure/large heart	1. |__| Yes<br>2. |__| No<br>9. |__| Does not know	|__|___| Age<br>***Does not know = 99 <u>or</u>***<br>|__|__|__|__| Year diagnosed<br>***Does not know = 9999***	1. |__|__| Hospitalized<br>2. |__|__| Surgery<br>3. |__|__| Medication<br>4. |__|__| No treatment
j. Stroke	1. |__| Yes<br>2. |__| No<br>9. |__| Does not know	|__|___| Age<br>***Does not know = 99 <u>or</u>***<br>|__|__|__|__| Year diagnosed<br>***Does not know = 9999***	1. |__|__| Hospitalized<br>2. |__|__| Surgery<br>3. |__|__| Medication<br>4. |__|__| No treatment
l. Liver Cirrhosis	1. |__| Yes<br>2. |__| No<br>9. |__| Does not know	|__|___| Age<br>***Does not know = 99 <u>or</u>***<br>|__|__|__|__| Year diagnosed<br>***Does not know = 9999***	1. |__|__| Hospitalized<br>2. |__|__| Surgry<br>3. |__|__| Medication<br>4. |__|__| No treatment
m. Chronic hepatitis	1. |__| Yes<br>2. |__| No<br>9. |__| Does not know	|__|___| Age<br>***Does not know = 99 <u>or</u>***<br>|__|__|__|__| Year diagnosed<br>***Does not know = 9999***	1. |__|__| Hospitalized<br>2. |__|__| Surgery<br>3. |__|__| Medication<br>4. |__|__| No treatment
n. Kidney failure	1. |__| Yes<br>2. |__| No<br>9. |__| Does not know	|__|___| Age<br>***Does not know = 99 <u>or</u>***<br>|__|__|__|__| Year diagnosed<br>***Does not know = 9999***	1. |__|__| Hospitalized<br>2. |__|__| Surgery<br>3. |__|__| Medication<br>4. |__|__| No treatment

3. Evaluation of co-morbidities:
   1. |__| Unsatisfactory
   2. |__| Questionable
   3. |__| Reliable

<u>MODULE OF HABITS: TOBACCO SMOKING</u>

The following questions are about the use of tobacco products. I will start by asking about the use of industrialized cigarettes.
Interviewer: Do not consider marijuana cigarettes or small cigars.

1. Are you a:

|____| 1. current daily or occasional smoker
|____| 2. ex-smoker
|____| 3. never smoked

<u>CURRENTLY DAILY AND OCCASIONAL SMOKER</u>

2. How old were you when you started smoking cigarettes regularly?

Interviewer: Does not know = 99
|___|___| Years

3. On an average, how may cigarettes do you smoke?

Interviewer: Record the amount and mark the frequency (day, week, or month). Should the response be in packages or cartons, ask how many in each and calculate the total number of cigarettes.

|__|__|__| Cigarettes per
Does not know = 999

1. |__| Day
2. |__| Week
3. |__| Month
9. |__| Does not know

4. Did you stop smoking cigarettes for a period of 1 year or more?

1. |__| Yes
2. |__| No ***(go to 12)***

5. Combining all the periods of time when you stopped smoking cigarettes, how long did you stop smoking?
Interviewer: Does not know: Years = 99
|__|__| Years

EX SMOKER

6. How old were you when you started smoking cigarettes regularly?
Interviewer: Does not know: Years = 99
|__|__| Years

7. How old were you when you stopped smoking cigarettes?
Interviewer: Does not know = 99 and go to 12; Otherwise go to section "ENVIRONMENTAL EXPOSURE TO CIGARETTE SMOKE.
|__|__| Years

8. How long ago did you stop smoking cigarettes?
Interviewer: Does not know: Years = 99; < 1 Year = 00
|__|__| Years

9. On the average, how many cigarettes did you smoke?
Interviewer: Record the amount and mark the frequency (day, week or month). Should the response be in packages or cartons, ask how many in each and calculate the total number of cigarettes.

|__|__|__| Cigarettes per
Does not know = 999

1. |__| Day
2. |__| Week
3. |__| Month
9. |__| Does not know

10. During the years you smoked cigarettes, did you stop smoking cigarettes for a period of time of 1 year or more?
    1. |__| Yes
    2. |__| No ***(go to 12)***

11. During the years you smoked cigarettes, combining all the periods of time you stopped smoking cigarettes, in total, how long did you stop smoking?
Interviewer: Does not know: Years = 99
|__|__| Years

Now I will ask you about cigarettes or small cigars made of tobacco that you put together in your home (not bought cigarettes).

	**Home-made cigarettes**
12. Do you smoke home-made cigarettes at least once per week?	1. |__| Yes<br>2. |__| No
13. How old were you when you started smoking at least one of these per week?	|__|__| Years<br>***Does not know = 99***
14. How old were you when you last smoked these cigarettes?	|__|__| Years<br>***Does not know = 99***<br>***Still smokes them = 96***
15. During the time you smoked them, on the average how many did you normally smoke per week?	|__|__| Cigarettes or small cigars/week<br>***Less than 1/Week = 00***<br>***Varied a lot = 96***<br>***Does not know = 99***
16. During the time you smoked these home-made cigarettes, was there a period of time when you stopped smoking them?	1. |__| Yes<br>2. |__| No
17. Combining all the periods of time you stopped smoking them, how long did you stop?	|__|__| Years<br>***Does not know = 99***

ENVIRONMENTAL EXPOSURE TO CIGARETTE SMOKE

18. At some time during your life, did anyone in your home, including individuals who worked there daily, smoke in your presence?
    1. |__| Yes
    2. |__| No
    3. |__| NC/does not remember

19. Of all the individuals who worked in your home, including those who worked daily in your home, how many individuals smoked in your presence in your home?
|__|__| individuals

20. What was your relationship with the individual who smoked?	21. How old were you when this individual smoked for the first time in your presence? ***Does not know: Years =99***	22. How old were you when this individual smoked for the last time in your presence? ***Does not know: Years =99***
24.1.________ |__|__|	*|__|__| Years*	*|__|__| Years*
24.2.________ |__|__|	*|__|__| Years*	*|__|__| Years*
24.3.________ |__|__|	*|__|__| Years*	*|__|__| Years*
24.4.________ |__|__|	*|__|__| Years*	*|__|__| Years*
24.5.________ |__|__|	*|__|__| Years*	*|__|__| Years*
24.6.________ |__|__|	*|__|__| Years*	*|__|__| Years*

23. Evaluation of Module:
1. |__| Unsatisfactory
2. |__| Questionable
3. |__| Reliable

MODULE OF HABITS: ALCOHOL CONSUMPTION

The following questions are about alcoholic consumption. We will consider one dose of alcoholic drink as one can of beer, one glass of wine, one drink, one dose of liquor or whiskey.

1. **During your whole life**, was there any period of time when you consumed at least one dose of alcoholic beverage such as beer, wine, whiskey, liquor, etc. per month for at least six months?
1. |__| Yes
2. |__| No ***(go to next module)***
9. |__| Does not know ***(go to next module)***

2. How old were you when you started drinking at least one dose of alcoholic beverage per month?
Interviewer: Does not know = 99
|__|__| Years

3. **During the last year** (last 12 months), what was the average number of doses of alcoholic beverages that you consumed?
1. |__| No dose or less than one per month
2. |__| 1 to 3 doses per month
3. |__| 1 dose per week
4. |__| 2 to 6 doses per week
5. |__| 7 to 13 doses per week (1 dose per day)
6. |__| 14 to 20 doses per week (2 doses per day)
7. |__| 21 to 28 doses per week (3 doses per day)
8. |__| 29 or more doses per week (4 or + doses per day)
9. |__| Does not know

4. How old were you when you stopped consuming at least one dose of alcoholic drink per month?
Interviewer: Do not consider periods of time when you consumed alcoholic beverages less than once per month.
Interviewer: Does not know = 99
|__|__| Years

5. How long ago did you stop consuming at least one dose of alcholic beverage per month?
Interviewer: Does not know: Years = 99; < 1 Year = 00
|__|__| Years

6. Evaluation of Module:
1. |__| Unsatisfactory
2. |__| Questionable
3. |__| Reliable

MODULE OF ANTHROPOMORPHIC FACTORS AND PHYSICAL ACTIVITY

1. How tall are you now?

Interviewer: Does not know, does not remember: 9,99

|__| , |__|__| meters

2. Currently, what is your approximate usual weight?

|__|__|__| . |__| |__| Kg

3. Not considering the times you were pregnant or nursing, what was your usual weight when you were...

Interviewees: When you do not remember, put 999,9

20 years old? |__|__|__| , |__| Kg
30 years old? |__|__|__| , |__| Kg
40 years old? |__|__|__| , |__| Kg
50 years old? |__|__|__| , |__| Kg
60 years old? |__|__|__| , |__| Kg
70 years old? |__|__|__| , |__| Kg

4. What was the most weight you have been? (not considering your weight during pregnancy)

Interviewees: When you do not remember, put 999,9

|__|__|__| , |__| Kg

5. How old were you when you weighed that much?

Interviewees: When you do not remember, put 999,9

|__|__| years

PHYSICAL ACTIVITY

1. Have you ever in your life done regular physical activities or exercise – that is, at least once per week, for 3 months or more?
   1. |__| Yes ***(go to 2)***
   2. |__| No ***(end of module)***

I would like for you to tell me about these activities and/or exercises, starting with those you did when you were younger.

	2. What physical activities or exercises have you performed or do you perform regularly?	3. What year did you start this activity?	4. What year did you stop this activity? ***Ongoing: 0000***	5. On the average, how many hours per week did you or do you perform this activity? ***Does not know: 99:99***
1)	________________________________|__|__| **(Code)**	|__|__|__|__|	|__|__|__|__|	|__|__|:|__|__| hours minutes
2)	________________________________|__|__| **(Code)**	|__|__|__|__|	|__|__|__|__|	|__|__|:|__|__| hours minutes
3)	________________________________|__|__| **(Code)**	|__|__|__|__|	|__|__|__|__|	|__|__|:|__|__| hours minutes
4)	________________________________|__|__| **(Code)**	|__|__|__|__|	|__|__|__|__|	|__|__|:|__|__| hours minutes
5)	________________________________|__|__| **(Code)**	|__|__|__|__|	|__|__|__|__|	|__|__|:|__|__| hours minutes

Physical Activity Code:

**Walk 01**	**Bike 04**	**Tennis 07**	**Dance 10**	**Hike 13**	**Football 16**
**Run 02**	**Swim 05**	**Bowl 08**	**Volleyball 11**	**Yoga 14**	**Martial Arts 17**
**Aerobics 03**	**Golf 06**	**Baseball 09**	**Soccer 12**	**Skate 15**	**Other 99 (Specify)**

6. Evaluation of module:
   1. |__| Unsatisfactory
   2. |__| Questionable
   3. |__| Reliable
