# Supplementary material for: Socioeconomic, Clinical, and Molecular Features of Breast Cancer Influence Overall Survival of Latin American Women
Source: Front Oncol. 2022 Mar 8;12:845527. doi: 10.3389/fonc.2022.845527 (PMC9071365; doi:10.3389/fonc.2022.845527)
Supplement: Supplementary file 1 [file DataSheet_1.zip › Supplementary Material/Supplementary Material 4.docx]

**SUPPLEMENTARY MATERIAL 4**

| **NCI - USLACRN Molecular Profile Breast Cancer Project -** IRBs approved for **Molecular Profiling of Stage II and III Breast Cancer in Latin American Women Receiving Standard-of-Care Treatment Study (NCI IRB-** 15CN055) | | |
| --- | --- | --- |
| Country | **Name of Committee** | **Approval ID** |
| Argentina | Comité de Etica en Investigación del Hospital Municipal de Oncología "María Curie" | N/A |
|  | Comité de Etica en Investigación del Instituto de Oncología "Angel Roffo" | N/A |
|  | Comité de Etica en Investigación del Hospital Interzonal General de Agudos "Eva Perón" | 35/13 |
|  | Comité de Docencia e Investigacion Hospital Thompson | 1/13 |
|  | Comité de Bioética Fundación Instituto Leloir | CBFIL-1A |
| Brasil | Comitê de Ética em Pesquisa do Instituto Nacional de Câncer (INCA), Rio de Janeiro, Brasil | CAAE 88713918.8.1001.5274 |
|  | Comitê de Ética em Pesquisa da Faculdade de Medicina da Universidade de São Paulo- Instituto de Câncer do Estado de São Paulo (ICESP), SP, Brasil | CAAE 88713918.8.1001.5274 |
|  | Comitê de Ética em Pesquisa do A.C.Camargo Cancer Center, SP, Brasil | CAAE 88713918.8.1001.5274 |
|  | Comitê de Ética do Hospital de Câncer de Barretos, SP, Brasil | CAAE 88713918.8.1001.5274 |
| Chile | CEC SSM Centro | N/A |
|  | CEC SSM Sur | N/A |
|  | CEC SSM Norte | N/A |
|  | CEC SSM Oriente | N/A |
| Mexico | Comisión Nacional de Investigación Ciéntífica del Instituto Mexicano del Seguro Social | Registro Nacional R-2011-785-035 |
|  | Comisión Federal para la Protección contra Riesgos Sanitarios (COFEPRIS) | R-COF164-196-198. |
|  | Comité de Investigación y Comité de Ética en Investigación del Hospital Civil de Guadalajara "Dr. Juan I. Menchaca" | R1043-10 |
|  | Comité de Ética, Enseñanza e Investigación del Instituto Jalisciense de Cancerología | 331/2010 |
|  | Comité de Etica en Investigación, Comité de Inverstigación y Comité deBioseguridad del Centro Universitario de Ciencias de la Salud, Universidad de Guadalajara | CI-01412 |
|  | Comisión de Bioética e Investigación, Universidad de Sonora | DMCS/CBIDMCS/D-10 |
|  | Comité de Ética, Secretaría de Salud Pública del Estado de Sonora | SSP-S-103/10 |
|  | Comisión Nacional de Investigación Científica, Instituto Mexicano del Seguro Social | 09-B5-61-2800/201100/1409, Registry number: R-2011-785-035 |
|  | Comité de Enseñanza e Investigación, Hospital General del Estado de Sonora “Dr. Ernesto Ramos Bours” | SSS-HGE-DM-2011-0192 |
|  | Comité de Ética, Hospital Infantil e Integral de la Mujer del Estado de Sonora | HIE 066/11, Registry number: 010/11 |
|  | Comité de Ética, Centro Estatal de Oncología (antes denominado Hospital Oncológico del Estado de Sonora) | SSS-HOES/EYC/2012/011 |
| Uruguay | Comité de Ética de la Investigación, Hospital de Clinicas "Dr. Manuel Quintela" | N/A |
|  | Comité de Ética, Dirección de Sanidad de las Fuerzas Armadas, Ministerio de Defensa Nacional | N/A |
|  | Comité de Ética en Investigación, Centro Hospitalario Pereira Rossell, Administración de los Servicios de Salud del Estado | N/A |
